# Generation of a new *Slc20a2* knockout mouse line as in vivo model for primary brain calcification

**DOI:** 10.1186/s13041-025-01240-8

**Published:** 2025-08-20

**Authors:** Hisaka Kurita, Hiroki Kitaura, Kazuya Nishii, Tomohiko Masaka, Kazuki Ohuchi, Masatoshi Inden, Akiyoshi Kakita, Masatake Osawa, Isao Hozumi

**Affiliations:** 1 Laboratory of Medical Therapeutics and Molecular Therapeutics, Department Biomedical Pharmaceutics, Gifu Pharmaceutical University, 1-25-4 Daigaku-nishi, Gifu City, 501-1196 Gifu Japan; 2 Department of Pathology, Brain Research Institute, Niigata University, 1- 757 Asahimachi, Chuo-ku, Niigata, 951-8585 Japan; 3 Department of Regenerative Medicine and Applied Medical Sciences, Graduate School of Medicine, Gifu University, Gifu City, Gifu Japan; 4 Department of Clinical Engineering, Faculty of Health Science, Komatsu University, Komatsu, Japan

**Keywords:** Primary brain calcification, *SLC20A2*, *In vivo* model

## Abstract

Primary brain calcification (PBC) is a neurodegenerative disease that causes bilateral ectopic calcification in the brain. In this study, using newly generated *Slc20a2* knockout (*Slc20a2*^−/−^) mice, we establish an in vivo model for PBC. In contrast to heterozygous *Slc20a2*^+/−^ mice (9/9 animals) showing no obvious abnormalities, the homozygous *Slc20a2*^*−/−*^ mice exhibited severe calcification at 11 months of age (5/5 animals). Whilst smaller in size and number, the deposits were also detectable in 5-month-old *Slc20a2*^−/−^ mice (2/2 animals). By contrast, no obvious alterations were detectable in visceral organs, including the lung, kidney, liver, and spleen. Consistently, in PBC patients, despite the systemic mineral metabolic disturbance, calcification occurs only in a brain restricted manner. Hence, these observations suggest that our mouse model is capable of recapitulating certain aspects of human PBC etiology. In summary, our data suggested the utility of an in vivo PBC mouse model in understanding the pathological mechanisms behind brain calcification, which leads in development of novel therapeutics against PBC.

**Supplementary Information:**

The online version contains supplementary material available at 10.1186/s13041-025-01240-8.

Patients with primary brain calcification (PBC) suffer from diverse clinical manifestations including Parkinsonism, cognitive disorder, and psychiatric symptoms. In the past decade, several causative gene mutations have been identified in *SLC20A2*, *PDGFRB* [1], *PDGFB* [2], *XPR1* [3], *MYORG* [4], *JAM2* [5], and *NAA60* [6], whereas the etiology of PBC still remains largely unknown. It has been previously reported that *Slc20a2* KO mice serve as a potential in vivo model for PBC [7]. In this report, we generated a new *Slc20a2* KO mouse line using the genome editing technology, CRISPR/Cas9, and evaluated its suitability as a PBC model.

To disrupt *Slc20a2* gene, we inserted a stop codon and an *MluI* site into the exon 3 sequences via the homology-directed repair mechanism mediated by Cas9/gRNA-induced DNA double strand break (DSB) following zygotic microinjection of ribonucleoprotein (Fig. 1A). The KO allele was confirmed by PCR genotyping and subsequent *MluI* digestion of PCR fragments (Fig. 1B). To check if any undesired off-target mutations were induced, we selected 14 potential DSB genomic locations using the Cas-OFFinder off-target prediction algorithm (http://www.rgenome.net/cas-offinder/) under the condition of satisfying up to 3 mismatches or 1 bulge plus up to 2 mismatches to the target sequence. As far as tested, we were unable to identify any mutations in these predicted off-target locations (Supplemental Table1 and Fig. 2). We used F2 mice in examinations of this study. The levels of Pit2 protein were diminished in *Slc20a2*^−/−^ mice compared with *Slc20a2*^+/+^ mice in kidney and cerebellum (Supplemental Fig. 1D), which were detected by using specific antibody for Pit2 used in the previous studies [8, 9]. Consistent with the reported *Slc20a2*^−/−^mice, our *Slc20a2*^−/−^ mice are smaller in size and show significantly decreased body weight, and the survival rate of *Slc20a2*^−/−^ mice was lower than that of the wild type (Supplemental Fig. 1G-I). At 11-months-old, whilst rarely detectable in *Slc20a2*^+/−^ mice (9/9 animals), the *Slc20a2*^−/−^ mice exhibited severe calcification (5/5 animals) in the brain. Consistent with previous reports [10, 11], calcium deposits in the *Slc20a2*^−/−^ mice were observed in the thalamus, hypothalamus, midbrain, pons, and cerebral cortex (Fig. 1C). The *Slc20a2*^−/−^ mice showed no detectable level of calcification in their visceral organs, including the liver, kidney, lung and spleen (Supplemental Fig. 1J).

**Fig. 1:**
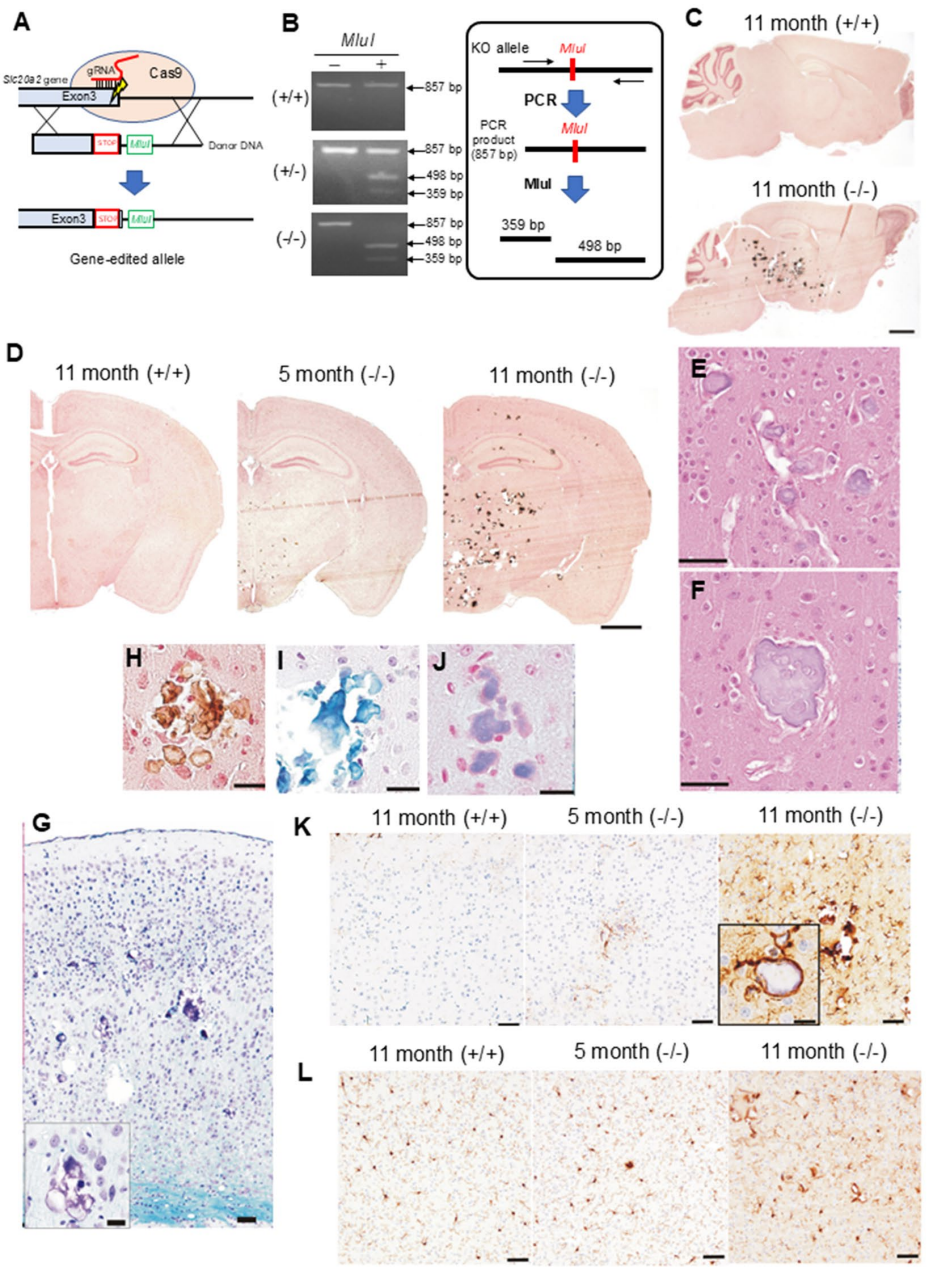
(**A**) Genomic editing strategy for the mouse *Slc20a2* gene was presented. (**B**) Genotyping of the *Slc20a2* knockout allele was determined. (**C**, **D**) Sagittal sections (**C**) and coronal sections of the brain at the hippocampus–amygdala level (**D**) using Kossa staining were performed. (**E**, **F**) Deposits near cortical vessels (**E**) and in the cortical parenchyma (**F**) in 11-month-old (−/−) mice were determined using HE staining. (**G**) Cerebral cortex using KB staining in 11-month-old (−/−) mice was performed. (**H**–**J**) Deposits stained using Kossa (**H**), Alcian Blue (**I**), and Berlin Blue stains in 11-month-old (−/−) mice (**J**) were performed. (**K**–**L**) Inflammatory changes in the cortex, glial fibrillary acidic protein (**K**), Iba-1 (**L**) were determined. Scale bars: **C**-**D**: 1 mm, **E**–**G**: 50 μm, (**G** inset), **H**–**J**: 2 μm, **K**-**L**: 50 μm

Compared to the mice in the previous paper, our mice are simple modifications that leave the original gene sequence as much as possible, so there is no insertion of reporter genes or drug resistance genes as in the previous report. The difference is that the possibility of phenotypic effect due to the insertion of a foreign gene for knockout is lower than that of the preceding mouse, which is an advantage of our mice. As we found slight reduction of *Slc20a2* mRNA levels in *Slc20a2*^+/−^ mice (Supplemental Fig. 1E) and Pit2 protein expression in *Slc20a2*^+/−^ mice was reduced to about 50% of that in *Slc20a2*^+/+^ mice (Supplemental Fig. 1F), the lack of calcification in the heterozygous mice doesn’t appear to be mediated by a dosage compensation effect, whereby defective protein and/or mRNA expression from the knockout allele might be compensated by the expression from wild-type allele. Given the reduction of *Scl20a2* expression, it is tempting to assume that the bulk phosphate transport activity of the cells expressing *Slc20a2* might be also decreased in the *Slc20a2*^+/−^ mice. If this would be the case, it is conceivable that it would require longer time period until calcification would become evident in the *Slc20a2*^+/−^ mice.

In the *Slc20a2*^−/−^ mice, deposits were observed mainly at the peri-vascular space and the brain parenchyma, where they formed concentric patterns (Fig. 1E and F). In contrast, neurons surrounding these deposits appeared to be unaffected (Fig. 1G). These deposits were positive not only for von Kossa, Alcian blue staining (for calcium detection) but also for Berlin blue staining (for iron detection) (Fig. 1H–J), suggesting the deposit formation may be resulting from defective homeostatic regulation of both calcium and iron ions in the brain.

Glial changes against the deposits were also observed in 11-month-old *Slc20a2*^−/−^ mice (Fig. 1K and L). In addition, some astrocytes take deposition into them reaching their process (Fig. 1K), and inflammatory changes appear to concomitantly progress gradually. These observations are consistent with the pathological abnormalities seen in PBC patients [12]. However, in sharp contrast with the previous report showing the presence of gliosis and its associated neuronal death in the brain autopsy samples of PBC patients [13], neither neurodegeneration, which is evident by the accumulation of phosphorylated tau and α-synuclein, (Supplemental Fig. 1K), nor neuronal destruction was detectable within the region surrounding the brain deposits in 11-month-old *Slc20a2*^−/−^ mice (Supplemental Fig. 1L). Although the pathological mechanism of neurodegeneration in PBC patients remained largely elusive, the fact that the *Slc20a2*^−/−^ mice exhibit severe calcification without showing obvious neurodegeneration suggests that calcification doesn’t represent a primary cause of neurodegeneration.

In conclusion, we successfully generated a new *Slc20a2* KO mouse model. This approach confirmed the utility of an in vivo PBC model to promote drug discovery research and unravel the molecular mechanisms behind brain calcification in humans.

## Supplementary Information

Below is the link to the electronic supplementary material.

Supplementary Material 1

Supplementary Material 2

Supplementary Material 3

## Data Availability

No datasets were generated or analysed during the current study.
