# Supplementary material for: Generation of a new Slc20a2 knockout mouse line as in vivo model for primary brain calcification
Source: Mol Brain. 2025 Aug 20;18:70. doi: 10.1186/s13041-025-01240-8 (PMC12369223; doi:10.1186/s13041-025-01240-8)
Supplement: Supplementary file 1 — Supplementary Material 1 [file 13041_2025_1240_MOESM1_ESM.docx]

**Supplemental information**

**Materials and methods**

**Generation of *Slc20a2* knockout mice**

Mouse studies were approved by the Animal Experiment Committee of Gifu Pharmaceutical University, Japan (Approval number: 2018-152) and the Committee for Animal Research and Welfare of Gifu University (Approval number: 2020-063). To generate *Slc20a2* KO mice, a donor DNA oligonucleotide containing a stop codon and an *MluI* site was inserted into exon 3 of the *Slc20a2* gene via homologous recombination, mediated by CRISPR/Cas9, as previously described [1]. Briefly, a fertilized mouse ovum was obtained from a mated female C57BL/6J mouse (Japan SLC, Inc., Hamamatsu, Japan), and super-ovulated by injection with pregnant mare serum gonadotropin (ASKA Animal Health Co., Tokyo, Japan) and human chorionic gonadotropin (ASKA Animal Health Co.). The zygote cytoplasm was micro-injected with 0.3-mM Cas9 protein (PNA Bio, Inc., Newbury Park, CA), 0.75-mM complementary RNA (crRNA), 0.75-mM trans-activating CRISPR RNA (Integrated DNA Technologies, Inc., Coralville, IA), and a homologous donor single-stranded DNA (ssDNA) spanning the *Slc20a2* exon 3 sequence, including a stop codon and an *MluI* site. Injected embryos were cultured overnight in KSOM medium (Merck KGaA, Darmstadt, Germany) to allow two-cell stage development. Embryos were transferred into the oviduct of pseudopregnant ICR females (Japan SLC, Inc.). The crRNA and ssDNA donor sequences were: *Slc20a2*-crRNA, 5′-GAGCUCGUCAAGAUUGGUAAGUUUUAGAGCUAUGCU-3′ and ssDNA donor, 5′-CCTCTAGCAAAGGAGAAGTGTATTGGCCAAAGGTAAAAGAAGACGCGTATTACCTATTTAGACGAGCTCCATCCACTGCACTCCTTTCGGGCCAATCGC-3′. Mice harboring the KO allele were selected in F0 generation and crossed with wild-type (wt) mice to confirm germline transmission of the KO allele. The heterozygous F1 KO mice were interbred to generate homozygous KO mice in the F2 generation. The body weights of F2 littermate mice, including wt (+/+), heterozygous (+/−), and homozygous (−/−) were measured from 4 to 14 weeks.

**Genotyping the *Slc20a2* KO allele using the *MluI* site**

DNA from mouse ear tissue was extracted for polymerase chain reaction (PCR). ExTaq DNA polymerase (Takara) was used for amplification, and reactions were amplified in a Veriti 96-well Thermal Cycler (Applied Biosystems) using the following conditions: 98°C for 10 s × 1 cycle; 98°C for 10 s, 68°C for 30 s, 72°C for 1 min × 35 cycles, and 72°C for 4 min × 1 cycle. Primers were: *Slc20a2-MluI*-forward; 5′-GGTGTGGCCCGGCTTCACAGC-3′ and *Slc20a2-MluI*-reverse; 5′-CCAGGAGGACTCATCACTAGC-3′. The PCR product was digested with *MluI* (Thermo Fisher) and electrophoresed on a 1% agarose gel containing ethidium bromide.

**Genotyping the *Slc20a2* KO allele using PCR primers for the wt and KO alleles**

For this procedure, DNA from mouse ear tissue was extracted and PCR-amplified, as described previously; the following amplification conditions were used: 98°C for 10 s ×1 cycle; 98°C for 10 s, 55°C for 30 s, 72°C for 1 min × 35 cycles, and 72°C for 4 min ×1 cycle. Primer sets were designed to detect the wt allele (*Slc20a2*-wt-forward and *Slc20a2*-wt-reverse primers) and the KO allele (*Slc20a2*-wt-forward and *Slc20a2*-ko-reverse primers) from the same DNA sample. Primers were: *Slc20a2*-wt-forward; 5′-GAGTGGCTACAACATGATTAG-3′, *Slc20a2*-wt-reverse; 5′-CCAAAGGTAAAAGAAGACGGC-3′, and *Slc20a2*-ko-reverse; 5′- CAAAGGTAAAAGAAGACGCGT-3′. The PCR product was electrophoresed as previously described.

S**equence analysis of the *Slc20a2* KO allele**

PCR was conducted using the following primer pair: *Slc20a2*-*MluI*-forward; 5′- GGTGTGGCCCGGCTTCACAGC-3′ and *Slc20a2-MluI*-reverse; 5′-CCAGGAGGACTCATCACTAGC-3′). The PCR fragment was purified and sub-cloned into the T-Vector pMD20 (Takara). DNA was then sequenced using the Big Dye Terminator v3.1 Cycle Sequencing kit (Applied Biosystems) and processed using a 3500 DNA Analyzer (Applied Biosystems).

**Off-target DNA sequence**

Off-target regions were predicted by Cas-OF Finder (<http://www.rgenome.net/cas-offinder/>) under the condition of satisfying 3 mismatches, or 1 bulge and 0 to 2 mismatches. Fourteen predicted regions were sequenced using the Big Dye Terminator v3.1 Cycle Sequencing kit and processed using a 3500 DNA Analyzer. Primers used are listed in supplemental table 2.

**Western blotting**

　Tissue samples from 4-month-old mice were homogenized in 10% SDS lysis buffer, and 20-30 µg of proteins were applied to SDS-PAGE and western blotting. The primary antibodies used for western blotting were as follows: mouse anti-Pit2 antibody (in-house, referred to in the previous papers [2, 3] ), and mouse anti-β-actin antibody (RID: AB_476692, 1:2000, Sigma Aldrich, St. Louis, MO, USA). The second antibodies were as follows: goat anti-mouse IgG antibody, peroxidase conjugated, H+L (RRID: AB_90456, 1:5000, Merck KGaA, Darmstadt, Germany), and anti-mouse IgG Kappa Light Chain, Recombinant Antibody, Horseradish Peroxidase (RRID: AB_2935617, 1:2000, Proteintech, Rosemont, IL, USA).

**Histopathology**

Twenty-three Slc20a2 mice (+/+: 7, +/−: 9, −/−: 7) were used for this part of the study. After perfused fixation, brains were removed and post-fixed in 4% paraformaldehyde/phosphate-buffered solution, dehydrated in a graded ethanol series, and embedded in paraffin wax. Serial sections were cut from the block (coronal sections of the cerebrum at the level of the hippocampus–amygdala and sagittal sections of the cerebellum). Hematoxylin and eosin and luxol fast blue staining was performed on sections. Immunostaining was also performed on sections (4-μm thick) using the following antibody selections: mouse monoclonal antibodies against rabbit polyclonal antibodies against glial fibrillary acidic protein (RRID:AB_10013382, Z0334, 1:1500; Dako, Glostrup, Denmark), Iba1 (RRID:AB_2665520, 1:500; Wako, Osaka, Japan), phosphorylated α-Synuclein (RRID: 015-25191, WAKO, Osaka, Japan) and phosphorylated Tau protein (RRID: 90206, CosmoBio, Tokyo, Japan). Immunolabeling was detected using a Histofine Simple Stain MAX-PO kit with a peroxidase–polymer-based secondary antibody (Nichirei Bio-sciences, Tokyo, Japan), and visualized using a diaminobenzidine/hydrogen peroxide solution. Sections were counterstained with hematoxylin. In some experiment, we treated decalicification with 10% EDTA and performed Bodian staining to observe neuronal fibers surrounding calicifications.

| **Supplemental table 1 Predicted off-target sequences in *Slc20a2* KO mice by Cas-OF Finder determined by sequence analysis** | | | | | | | |
| --- | --- | --- | --- | --- | --- | --- | --- |
| **#Bulge type** | **crRNA** | **predicted Off-Target DNA sequences** | **Chromosome** | **Position** | **Direction** | **Mismatches** | **Bulge Size** |
| X | GAGCTCGTCAAGATTGGTAANGG | GAaCTCaTCAAGATTGGTAcAGG | chr17 | 60935526 | + | 3 | 0 |
| X | GAGCTCGTCAAGATTGGTAANGG | GAGCTCGgCAAGATgGGTtATGG | chr11 | 38835867 | + | 3 | 0 |
| X | GAGCTCGTCAAGATTGGTAANGG | GAGCTCcTgAAGATgGGTAATGG | chr3 | 141146896 | + | 3 | 0 |
| RNA | GAGCTCGTCAAGATTGGTAANGG | GAGCTC-TCtAGATTGGTcATGG | chr12 | 45284214 | - | 2 | 1 |
| DNA | GAGCTCGTCAAGA-TTGGTAANGG | GAGCgaGTCAAGACTTGGTAAAGG | chr4 | 65797471 | + | 2 | 1 |
| RNA | GAGCTCGTCAAGATTGGTAANGG | GAGCT-GgCAAGATgGGTAAGGG | chr6 | 56735730 | - | 2 | 1 |
| RNA | GAGCTCGTCAAGATTGGTAANGG | GAGCTC-TCAAGATTGGgtAAGG | chr9 | 28129117 | + | 2 | 1 |
| RNA | GAGCTCGTCAAGATTGGTAANGG | GAGCTgGT-gAGATTGGTAAAGG | chrX | 160750666 | + | 2 | 1 |
| RNA | GAGCTCGTCAAGATTGGTAANGG | cAGCT--TaAAGATTGGTAAGGG | chr1 | 53017122 | - | 2 | 2 |
| RNA | GAGCTCGTCAAGATTGGTAANGG | GAGCTCtTCAgG--TGGTAAAGG | chr1 | 61419661 | + | 2 | 2 |
| RNA | GAGCTCGTCAAGATTGGTAANGG | GAGCTC--CAAGAaTGtTAAGGG | chr3 | 104555268 | + | 2 | 2 |
| RNA | GAGCTCGTCAAGATTGGTAANGG | GAGCT--TCAAGAaTGGTgAGGG | chr3 | 108811978 | - | 2 | 2 |
| RNA | GAGCTCGTCAAGATTGGTAANGG | GtGCTCGTCAAGAT--GTAtGGG | chr4 | 87053729 | + | 2 | 2 |
| RNA | GAGCTCGTCAAGATTGGTAANGG | aAGCTC--CAAGcTTGGTAATGG | chr7 | 135695712 | - | 2 | 2 |

| **Supplemental table 2 Primers used for sequence analysis of off-target regions** | | |
| --- | --- | --- |
| Position |  | sequences (5' to 3') |
| chr17 60935526 | Forward | TCTCTATTTTGGATCTTTGTTTTCG |
|  | Reverse | AAATGGGTACCAGTTAACACCACTA |
| chr11 38835867 | Forward | GCAAGTATTGGTAAGTGACAAACTG |
|  | Reverse | AAGCAAACATAACATGTAACAAGCA |
| chr3 141146896 | Forward | AGTGTTATGGACTCAAACAGATGAA |
|  | Reverse | TCTAATTGGTGAGGAAAGGAGACTA |
| chr12 45284214 | Forward | GCTAAGCTTACATCTTGTGAAAAGG |
|  | Reverse | AAGGCTGAATGAAGAACAAACATAA |
| chr4 65797471 | Forward | AAGTAAAAGACACTTCTGGTTCCAA |
|  | Reverse | ATGTTCAGTAGCTCTTGTATCATGC |
| chr6 56735730 | Forward | CTTCTCCTTCTCTTACTCCCAGTCT |
|  | Reverse | TGAACTAAAACCAGTACTCCCAAAG |
| chr9 28129117 | Forward | TCTTGAATTTCATTCTCTTTGAAGG |
|  | Reverse | TGAGATTAGGATTTTTCAGCTTTGT |
| chrX 160750666 | Forward | AAAACAGAGCACTGGAGAACTAAGA |
|  | Reverse | TGCTTTAATGTCTGTGTACAGGGTA |
| chr1 53017122 | Forward | GGAGTTGTGCTGTTTTAGGAATCTA |
|  | Reverse | CAACTTGATCCACTCCTGTCTCTAT |
| chr1 61419661 | Forward | CTTTGAACTTTCTGTCTGTGTCCTT |
|  | Reverse | CCAGCAAATTTTAAACTTATTTGAAG |
| chr3 104555268 | Forward | TAGAATACTCAAAACAGGAGGCAAG |
|  | Reverse | TGGAAATTATATGCGTTGTTTCTTT |
| chr3 108811978 | Forward | AAAATAATGCAATGTAGCCAGAGAC |
|  | Reverse | AAGTTAAATGCAGTCCCATGAAATA |
| chr4 87053729 | Forward | TGATGACTGTCTTTTGCTAGTGAAC |
|  | Reverse | TCTGGTCCATACTTTACACTGAACA |
| chr7 135695712 | Forward | TATTGTTAGCTCAGAAATTGGAACC |
|  | Reverse | CTCTTCTTTTACACCATCGTTGATT |

**Legends of supplemental figure 1**

(A) A schematic of crossing experiments to generate *Slc20a2* (−/−) mice was presented. (B) Genotyping the *Slc20a2* KO allele using primer sets to detect the wild-type and KO alleles was presented. (C) Sequence analysis for the *Slc20a2* KO allele was performed. (D) Pit2 protein levels were determined in the kidney and cerebellum from 4-month-old mice. Lysis samples of intact HEK293T cells, and HEK293T cells transfected with pCMV-IRES-AcGFP vector (mock) or pCMV-mPit2-HA-IRES-AcGFP vector (positive control: PC) were also loaded. (E) *Slc20a2* mRNA level was determined in brain. (F) Pit2 protein levels were determined in the cerebellum of all *Slc20a2* genotypes. N.D.=not detected. (G-I) Body weights for *Slc20a2* KO mice were determined. (I) The survival curve of *Slc20a2* KO mice was presented. (J) Deposits in visceral organs from an 11-month-old *Slc20a2* (−/−) mouse; the same mouse is also shown in Fig. 1C and 1D were determined. (K) Immunostaining of phosphorylated Tau (pTau (AT8)) and phosphorylated α-Synuclein (α-Syn (pNACP)) in 11-month-old *Slc20a2* (−/−) mouse (L) Bodian staining in 11-month-old *Slc20a2* (−/−) mouse were performed.

**Legends of supplemental figure 2**

Sequence data of off-target regions were presented.

**References**

1. Kakuda K, Niwa A, Honda R, Yamaguchi KI, Tomita H, Nojebuzzaman M, Hara A, Goto Y, Osawa M, Kuwata K: **A DISC1 point mutation promotes oligomerization and impairs information processing in a mouse model of schizophrenia**. *J Biochem* 2019, **165**(4):369-378.

2. Cheng X, Zhao M, Chen L, Huang C, Xu Q, Shao J, Wang HT, Zhang Y, Li X, Xu X *et al*: **Astrocytes modulate brain phosphate homeostasis via polarized distribution of phosphate uptake transporter PiT2 and exporter XPR1**. *Neuron* 2024, **112**(18):3126-3142 e3128.

3. Zhao M, Cheng X, Chen L, Zeng YH, Lin KJ, Li YL, Zheng ZH, Huang XJ, Zuo DD, Guo XX *et al*: **Antisense oligonucleotides enhance SLC20A2 expression and suppress brain calcification in a humanized mouse model**. *Neuron* 2024, **112**(19):3278-3294 e3277.
