# Supplementary figures and images for: Generation of a new Slc20a2 knockout mouse line as in vivo model for primary brain calcification
Source: Mol Brain. 2025 Aug 20;18:70. doi: 10.1186/s13041-025-01240-8 (PMC12369223; doi:10.1186/s13041-025-01240-8)

# Supplemental figure 1

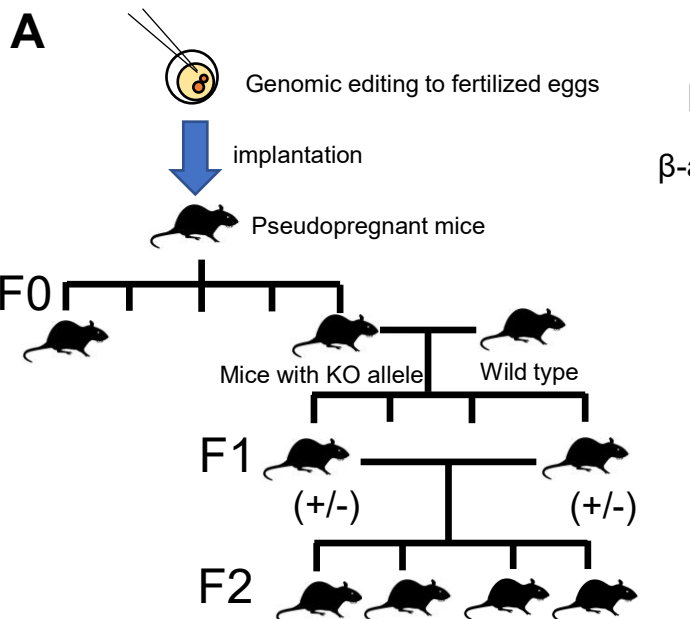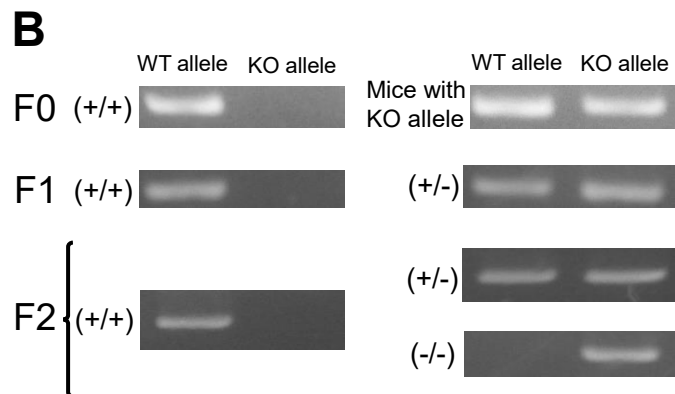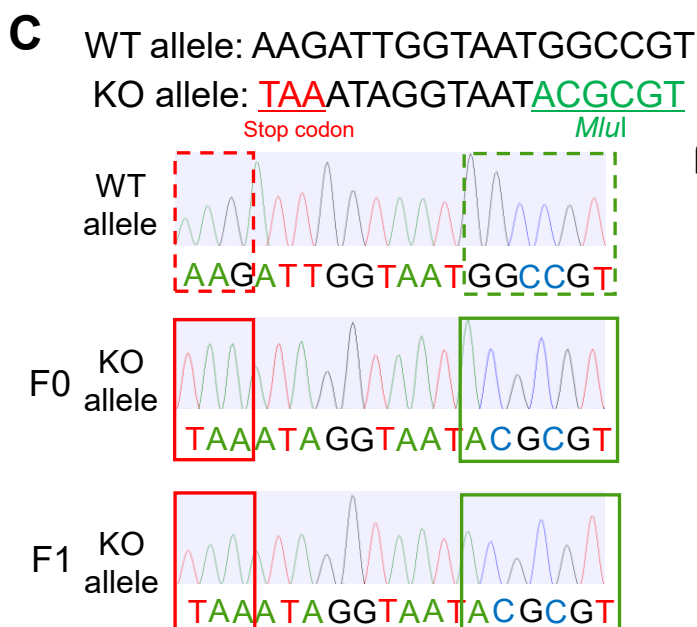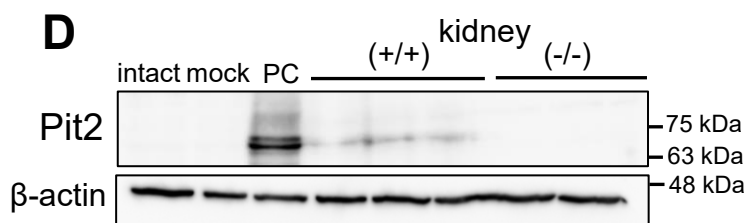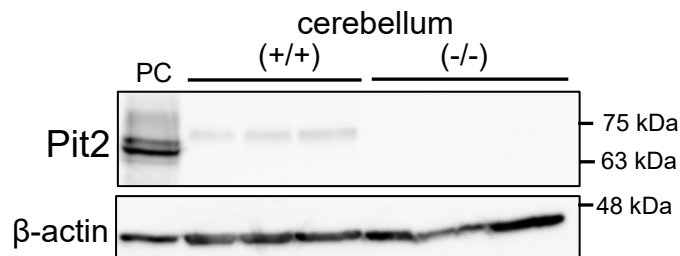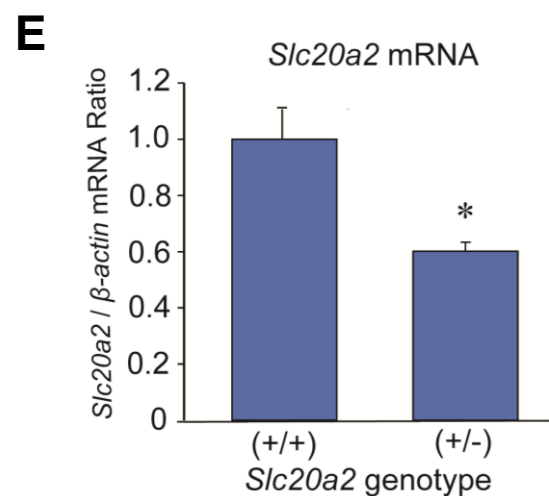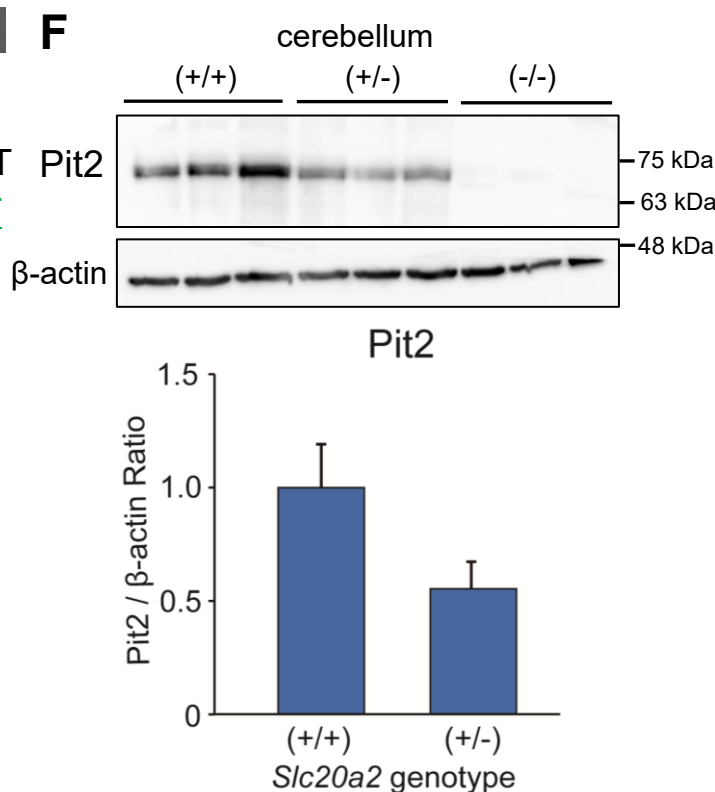

Supplemental figure 1

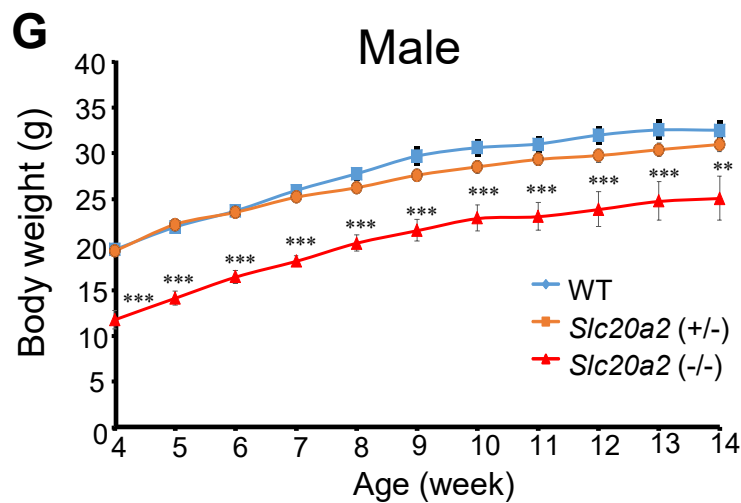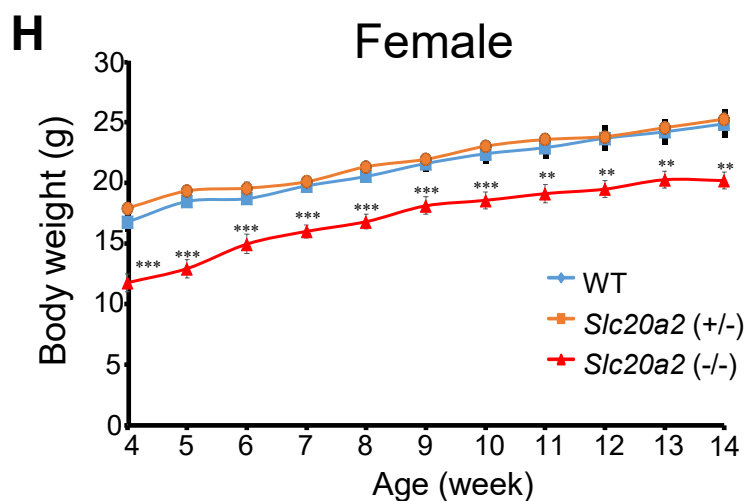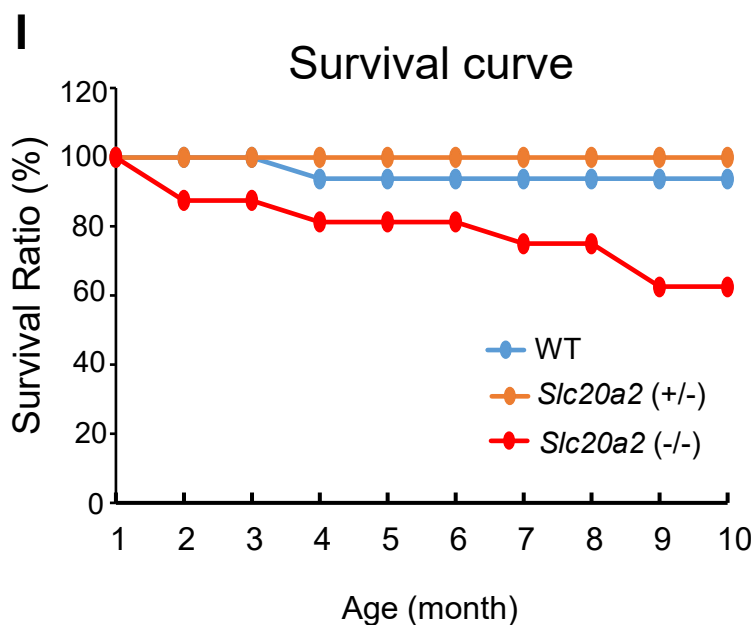

# Supplemental figure 1

**J**

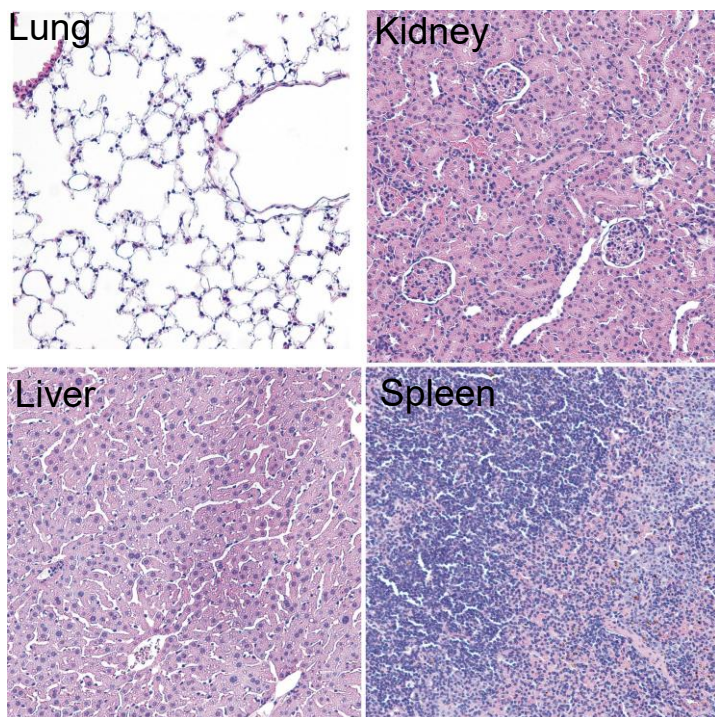

**K**

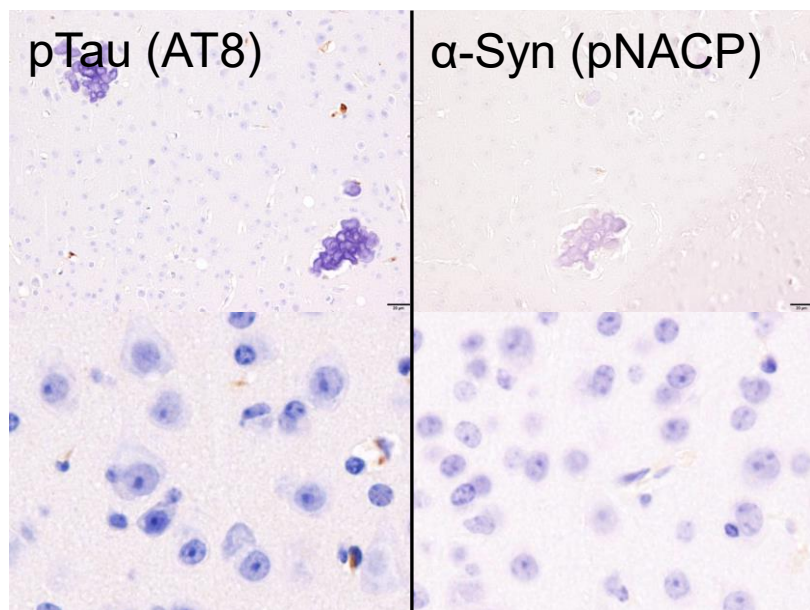

**L**

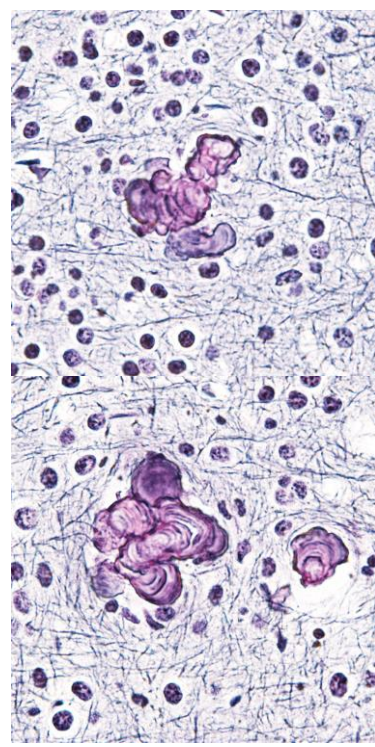

20  $\mu$ m

Supplement: Supplementary file 2 — Supplementary Material 2 [file 13041_2025_1240_MOESM2_ESM.pdf]
