# Supplementary material for: Generation of a new Slc20a2 knockout mouse line as in vivo model for primary brain calcification
Source: Mol Brain. 2025 Aug 20;18:70. doi: 10.1186/s13041-025-01240-8 (PMC12369223; doi:10.1186/s13041-025-01240-8)

Supplemental figure 2

Chr17 60935526

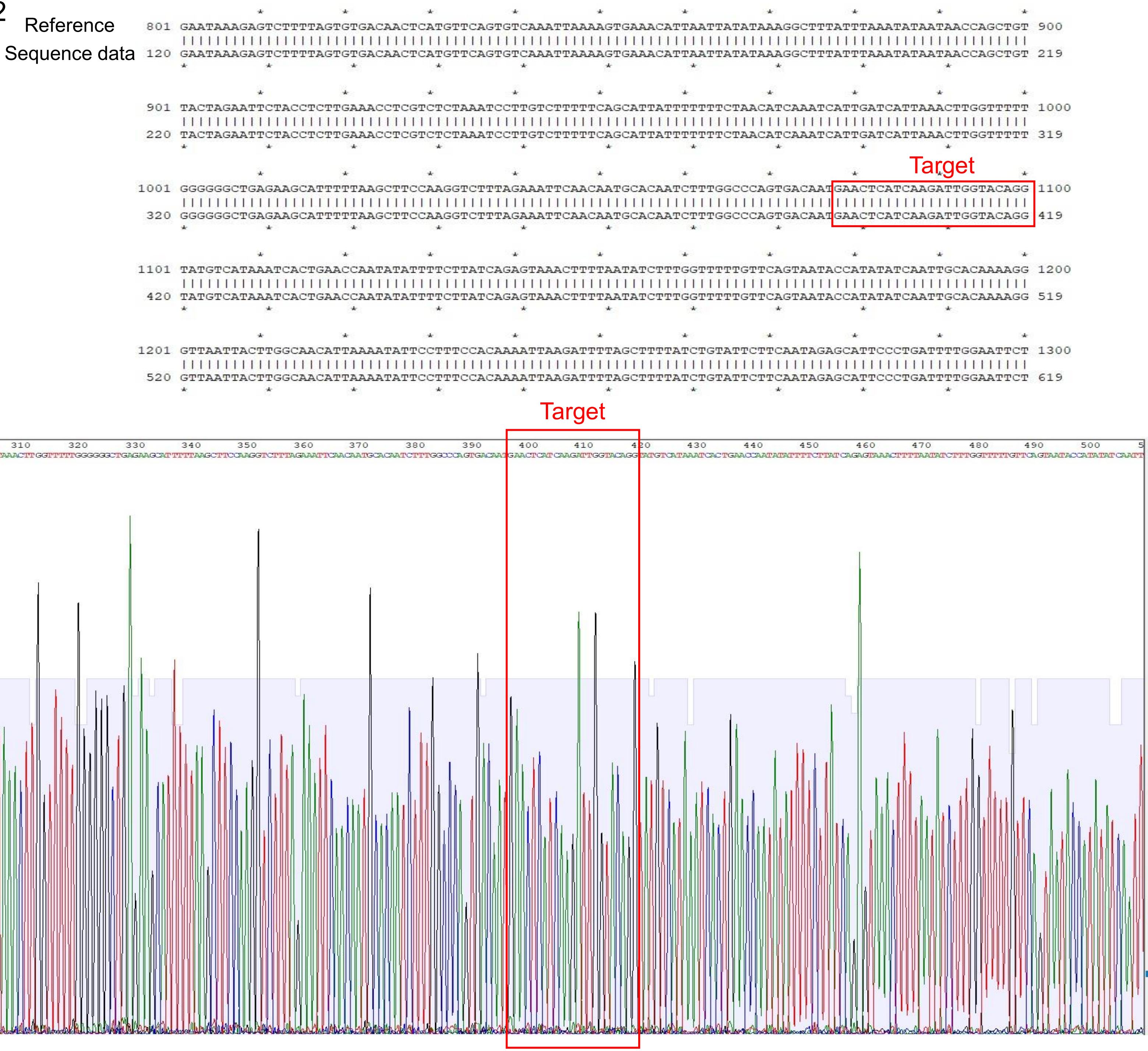

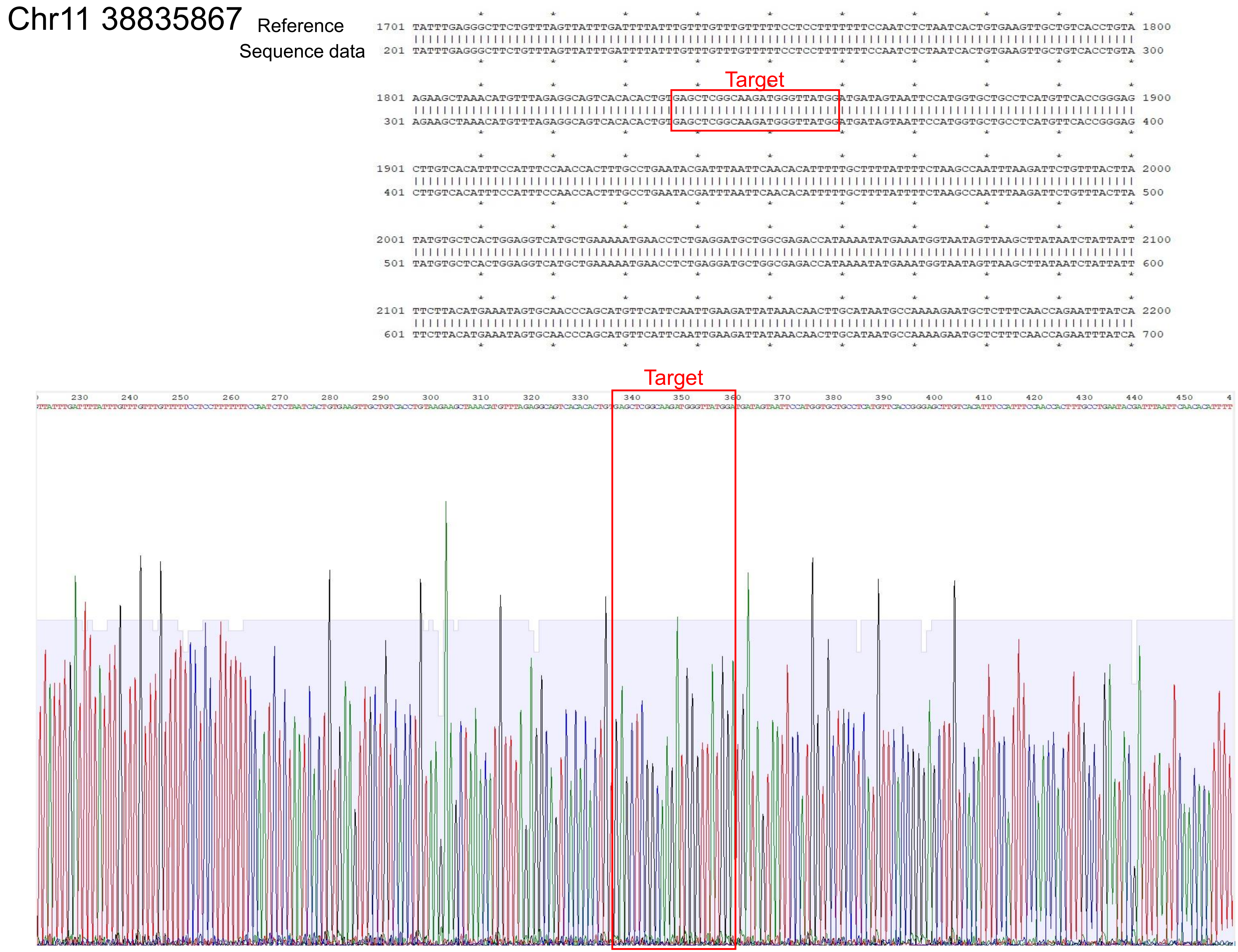

Chr11 38835867
Reference
Sequence data
Target
Target

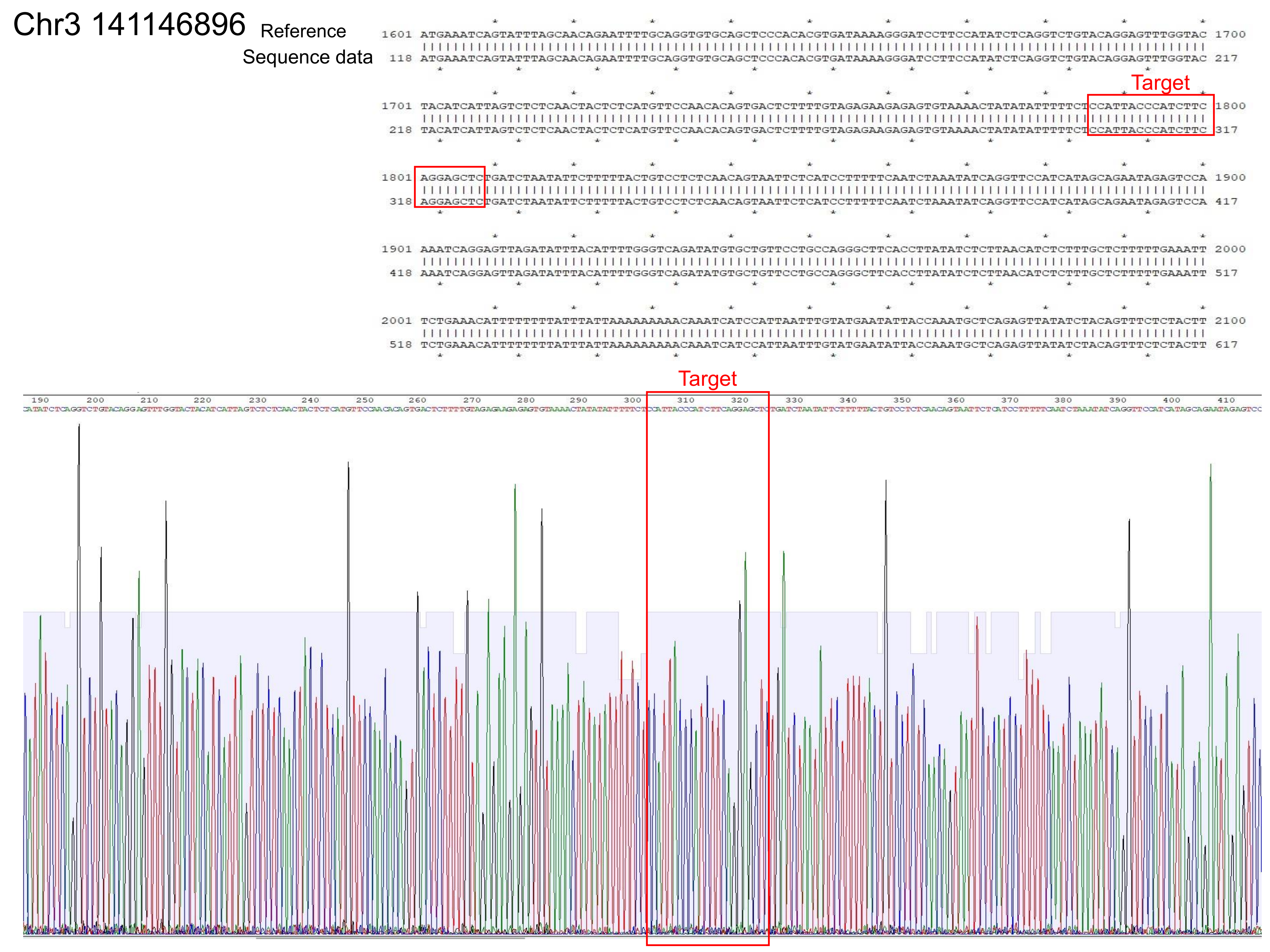
Chr3 141146896
Reference
Sequence data
1601 ATGAAATCAGTATTTAGCAACAGAATTTTGCAGGTGTGCAGCTCCCACACGTGATAAAAGGGATCCTTCCATATCTCAGGTCTGTACAGGAGTTTGGTAC 1700
118 ATGAAATCAGTATTTAGCAACAGAATTTTGCAGGTGTGCAGCTCCCACACGTGATAAAAGGGATCCTTCCATATCTCAGGTCTGTACAGGAGTTTGGTAC 217
Target
1701 TACATCATTAGTCTCTCAACTACTCTCATGTTCCAACACAGTGACTCTTTTGTAGAGAAGAGAGTGTAAAACTATATATTTTTCTCCATTACCCATCTTC 1800
218 TACATCATTAGTCTCTCAACTACTCTCATGTTCCAACACAGTGACTCTTTTGTAGAGAAGAGAGTGTAAAACTATATATTTTTCTCCATTACCCATCTTC 317
1801 AGGAGCTCTGATCTAATATTCTTTTTACTGTCCTCTCAACAGTAATTCTCATCCTTTTTCAATCTAAATATCAGGTTCCATCATAGCAGAATAGAGTCCA 1900
318 AGGAGCTCTGATCTAATATTCTTTTTACTGTCCTCTCAACAGTAATTCTCATCCTTTTTCAATCTAAATATCAGGTTCCATCATAGCAGAATAGAGTCCA 417
1901 AAATCAGGAGTTAGATATTTACATTTTGGGTCAGATATGTGCTGTTCCTGCCAGGGCTTCACCTTATATCTCTTAACATCTCTTTGCTCTTTTTGAAATT 2000
418 AAATCAGGAGTTAGATATTTACATTTTGGGTCAGATATGTGCTGTTCCTGCCAGGGCTTCACCTTATATCTCTTAACATCTCTTTGCTCTTTTTGAAATT 517
2001 TCTGAAACATTTTTTTTATTTATTAAAAAAAAACAAATCATCCATTAATTTGTATGAATATTACCAAATGCTCAGAGTTATATCTACAGTTTCTCTACTT 2100
518 TCTGAAACATTTTTTTTATTTATTAAAAAAAAACAAATCATCCATTAATTTGTATGAATATTACCAAATGCTCAGAGTTATATCTACAGTTTCTCTACTT 617
Target
190 200 210 220 230 240 250 260 270 280 290 300 310 320 330 340 350 360 370 380 390 400 410

Chr12 45284214

```
Reference      CTAAATTTACTATGTGTGTATGATTATACATGAAGAGTGGCATCATGGTAAGCATTATTATGACCTAGTTATTGTTTTCATTTGGACATAA 1400
Sequence data  CTAAATTTACTATGTGTGTATGATTATACATGAAGAGTGGCATCATGGTAAGCATTATTATGACCTAGTTATTGTTTTCATTTGGACATAA 108

1401 GGAAATATGATTATGTATCAAGTTGACAAGGGATGGATTATGATGGATGTTGTTCATATATGTCAACTTGACTATATATGGAATGAACTACAGTTCAGAA 1500
 109 GGAAATATGATTATGTATCAAGTTGACAAGGGATGGATTATGATGGATGTTGTTCATATATGTCAACTTGACTATATATGGAATGAACTACAGTTCAGAA 208

1501 ATAGAGGACTGCCAAGAGCAAGCCTTGGCTAGTTTAGGTTCCTGAGAGAAGGGGTGTTTGAGTGTGTTGAAACAAACTTAAACACAAAGTTAAATGGTGG 1600
 209 ATAGAGGACTGCCAAGAGCAAGCCTTGGCTAGTTTAGGTTCCTGAGAGAAGGGGTGTTTGAGTGTGTTGAAACAAACTTAAACACAAAGTTAAATGGTGG 308

                                                 Target
1601 GTCCAAGCTGGCAGCTTGTGCTTAAGGTGTCTCCGTAGAGAGCTCTCTAGATTGGTCATGGCTCTGTAGTCTTCTGGGGATAGCTTTTCAGATTGCTTCT 1700
 309 GTCCAAGCTGGCAGCTTGTGCTTAAGGTGTCTCCGTAGAGAGCTCTCTAGATTGGTCATGGCTCTGTAGTCTTCTGGGGATAGCTTTTCAGATTGCTTCT 408

1701 CTCTCTCTCTCTCTCTCTCTCTCTCTCTGTGTGTGTGTGTGTGTGCTCTGATCTGCTC
 409 CTCTCTCTCTCTCTCTCTCTCTCTCTCTGTGTGTGTGTGTGTGTGCTCTGATCTGCTC
```

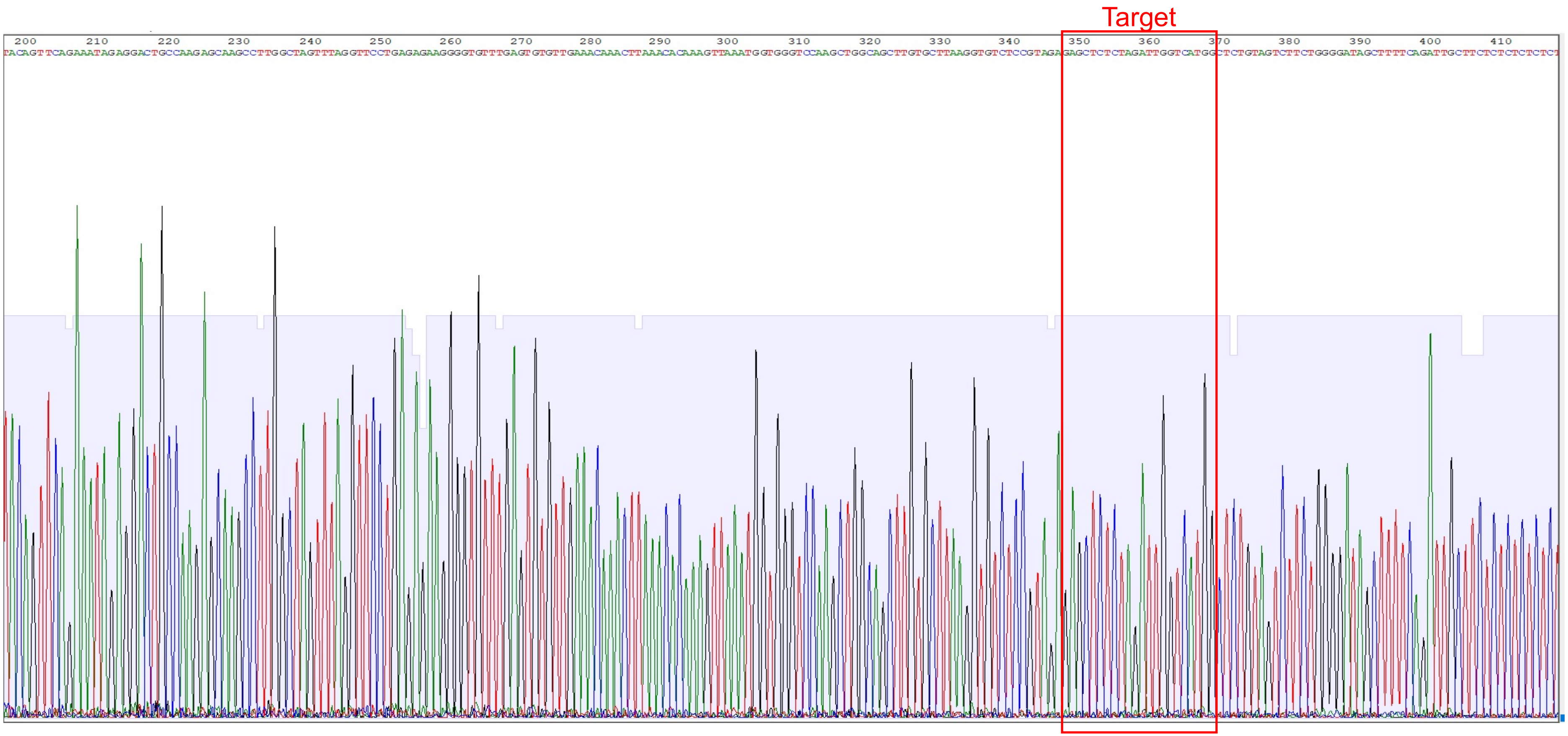

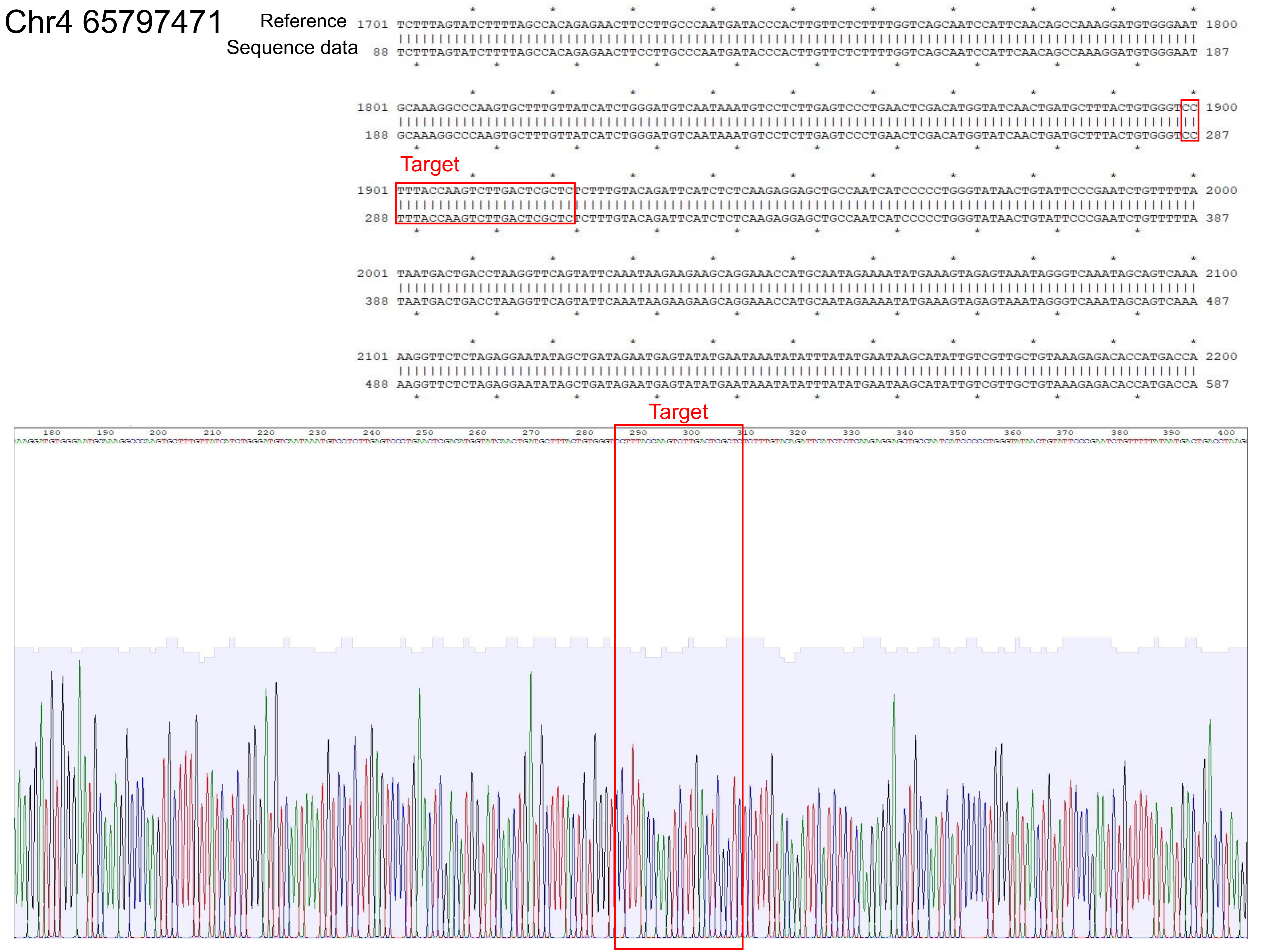
Chr4 65797471
Reference
Sequence data
Target
Target

Chr6 56735730

Reference
Sequence data

```
1801 TGAATAGATTGGTGGGTGCACTAATGACTGTGTTATCCCTTTTTCCAGGTAAGAGGATAACCATTACTACTTTTGCTTATTGTTATTGAAATTTTGACCT 1900
 294 TGAATAGATTGGTGGGTGCACTAATGACTGTGTTATCCCTTTTTCCAGGTAAGAGGATAACCATTACTACTTTTGCTTATTGTTATTGAAATTTTGACCT 393

1901 TTTAATAATAAAAGTATACAAAAAGTAAACTCATTAGGGTTAATATTTCCATTTTAATTTTCCTTTTGGCAATATTTTTTTTTCCTTTAGCCTGGTTTTC 2000
 394 TTTAATAATAAAAGTATACAAAAAGTAAACTCATTAGGGTTAATATTTCCATTTTAATTTTCCTTTTGGCAATATTTTTTTTTCCTTTAGCCTGGTTTTC 493

2001 TTTCCTTATTTTAATTTTAACATTGGGGAGAGGGATGGTGTAAGCCATGGCATGCTGGTGGTCAGAGGACAACCTGTGAGAGTGTACCCCCCATTGTGTG 2100
 494 TTTCCTTATTTTAATTTTAACATTGGGGAGAGGGATGGTGTAAGCCATGGCATGCTGGTGGTCAGAGGACAACCTGTGAGAGTGTACCCCCCATTGTGTG 593

                                                          Target
2101 TTGTGGTATTCAACCTAGGTTGTCAGGTTTGGCTTGGCAACAAGCACCCTTACCCATCTTGCCAGCTCCCTTTTTGTTTGTTTGTTTGTTTGTTTGTTTC 2200
 594 TTGTGGTATTCAACCTAGGTTGTCAGGTTTGGCTTGGCAACAAGCACCCTTACCCATCTTGCCAGCTCCCTTTTTGTTTGTTTGTTTGTTTGTTTGTTTC 693

2201 AATTTTTTATTAGATATTTTCTTCATTTACATTTCAAATGCGATCCCGAAAGTCCCCTATACCCTCCCCCTGCCCTGCTCCCCTACCTACCCATTCCCAC 2300
 694 AATTTTTTATTAGATATTTTCTTCATTTACATTTCAAATGCGATCCCGAAAGTCCCCTATACCCTCCCCCTGCCCTGCTCCCCTACCTACCCATTCCCAC 793
```

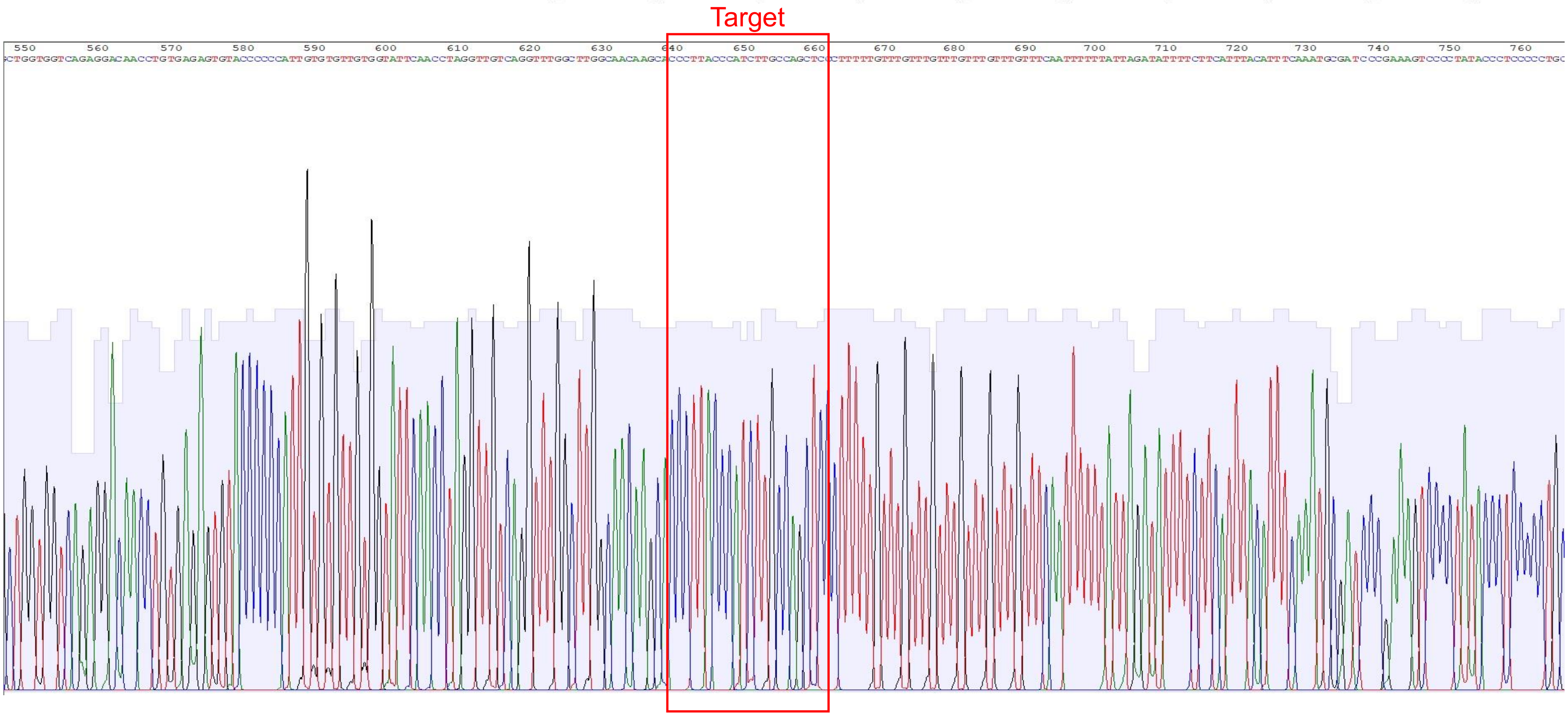

Chr9 28129117

Reference / Sequence data

```
Reference      1601 CCCTGACACAGTAGGTCACTTGTGGAATATTAGCCTGAGGGCTCTAGAAGCAGGGAAAGGGGCAGAGGCATTCCAGGGTGAAAGGAACAGCTCCTTGCAA 1700
Sequence data   192 CCCTGACACAGTAGGTCACTTGTGGAATATTAGCCTGAGGGCTCTAGAAGCAGGGAAAGGGGCAGAGGCATTCCAGGGTGAAAGGAACAGCTCCTTGCAA 291

               1701 GATTGTAAGTGGTGGTTGAGATGGATGGGGGCCCTTGGGAAGGGAAAGTGTCACAAGTGTCAAGGGTAAGTGGTGCAATTAAGAGTGTACTTTATGTGGT 1800
                292 GATTGTAAGTGGTGGTTGAGATGGATGGGGGCCCTTGGGAAGGGAAAGTGTCACAAGTGTCAAGGGTAAGTGGTGCAATTAAGAGTGTACTTTATGTGGT 391

               1801 CCTGCTTGGCCAGGTTATATATGGCTGTTTGGTTAGAGAGAGCTCTCAAGATTGGGTAAGGGGTTATCAACATTGAGTGAGGGGATCTTAGATAGTCAGG 1900
                392 CCTGCTTGGCCAGGTTATATATGGCTGTTTGGTTAGAGAGAGCTCTCAAGATTGGGTAAGGGGTTATCAACATTGAGTGAGGGGATCTTAGATAGTCAGG 491

               1901 GAGGGAGGGTTGTATACCTGGTTCTACATTTGGCTGCACATGCTGTTTAGGCTAAAGAGGGCTCATAGGTCAGGGTATATAGATCACAGATATTCAAAGA 2000
                492 GAGGGAGGGTTGTATACCTGGTTCTACATTTGGCTGCACATGCTGTTTAGGCTAAAGAGGGCTCATAGGTCAGGGTATATAGATCACAGATATTCAAAGA 591

               2001 GGAGAATGGCCTTACTGGTCTTGTTAGGCTAGGCCACATGTCCAGTCCAACTTCTTACATTAAATGCAATCTGGATCAAATTCTCATTTTTCAAACAGCA 2100
                592 GGAGAATGGCCTTACTGGTCTTGTTAGGCTAGGCCACATGTCCAGTCCAACTTCTTACATTAAATGCAATCTGGATCAAATTCTCATTTTTCAAACAGCA 691
```

Target: GAGCTCTCAAGATTGGGTAAGG

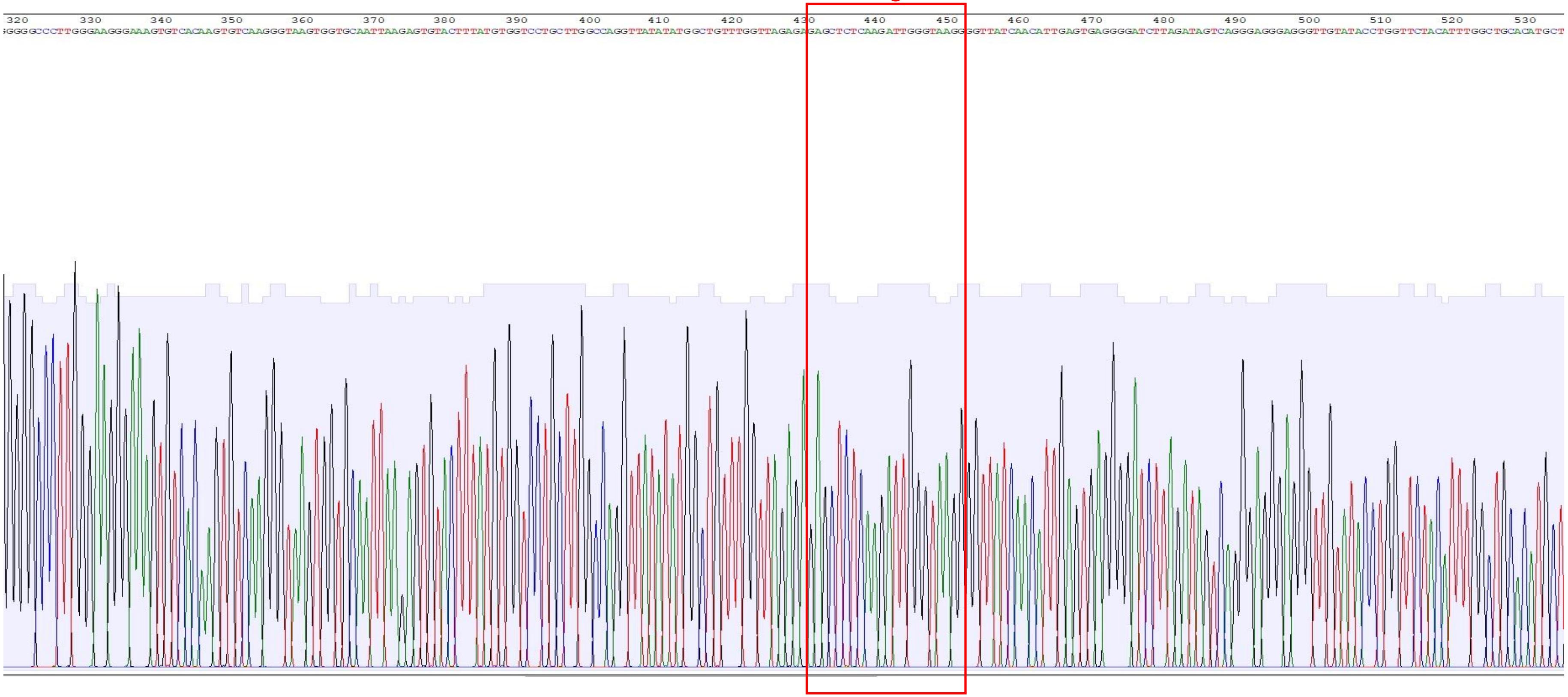

ChrX 160750666

Reference / Sequence data

```
1201 GTATCGCTACAATCTGTTCCTTTATCCTGTAAGTGCTTACATTTTCTGCATATTTCAGTTTGACATCTAGAATATGAGGCAATGGCAACTCTCTATTTCT 1300
 266 GTATCGCTACAATCTGTTCCTTTATCCTGTAAGTGCTTACATTTTCTGCATATTTCAGTTTGACATCTAGAATATGAGGCAATGGCAACTCTCTATTTCT 365

1301 TCTCATTTTCCTTCTGGAGGAGCCAAAATCTGCCATTCCAAGACAGAAGTGAAACAAAGCTGATATGGGAATACGTGTCCCAGTCATGTGAAGGCACAAG 1400
 366 TCTCATTTTCCTTCTGGAGGAGCCAAAATCTGCCATTCCAAGACAGAAGTGAAACAAAGCTGATATGGGAATACGTGTCCCAGTCATGTGAAGGCACAAG 465

1401 GGCAGAAGTGACTTAAGCATAGATGCCCAAGTAGCACTACAAGGCCCTCATGACATTCTTGAACTTGTGCTACGTGAGGCTACTCTGCTCCTTCAGACTC 1500
 466 GGCAGAAGTGACTTAAGCATAGATGCCCAAGTAGCACTACAAGGCCCTCATGACATTCTTGAACTTGTGCTACGTGAGGCTACTCTGCTCCTTCAGACTC 565

                                                               Target
1501 CTGTAGGAAAGACCTCAGACATCTCCAATTCGGAAGAGTGAAGAAACAGGCCTAGAGCTGGTGAGATTGGTAAAGGCCTATGCTGCCAAACCTGATGACC 1600
 566 CTGTAGGAAAGACCTCAGACATCTCCAATTCGGAAGAGTGAAGAAACAGGCCTAGAGCTGGTGAGATTGGTAAAGGCCTATGCTGCCAAACCTGATGACC 665

1601 TGAGTTTAATTCCCAAGATCCACATGGTAAAAAGAGGAGCCAACTCCCACAAGTTGTCCTCTGACTTCCACTCATGGTGCTATGGCAG
 666 TGAGTTTAATTCCCAAGATCCACATGGTAAAAAGAGGAGCCAACTCCCACAAGTTGTCCTCTGACTTCCACTCATGGTGCTATGGCAG
```

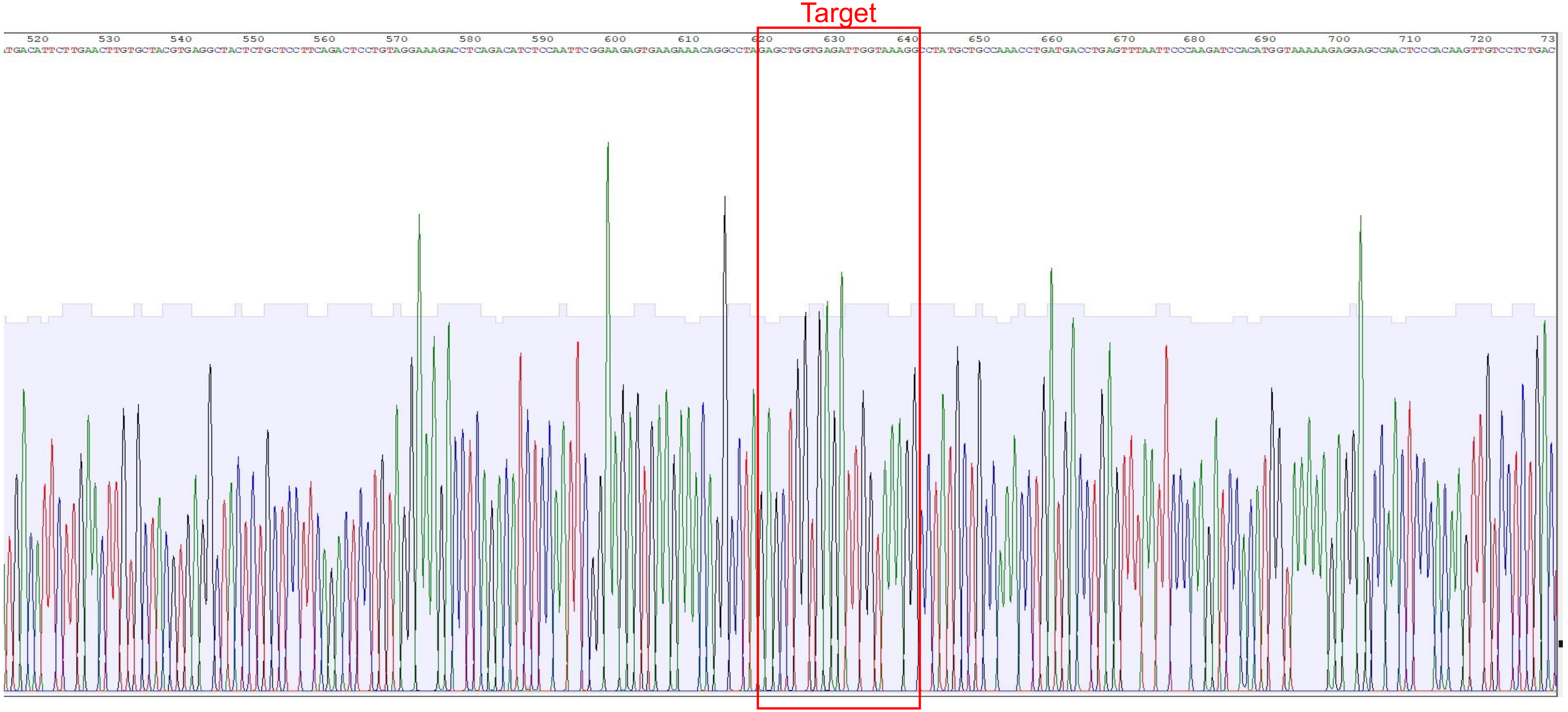

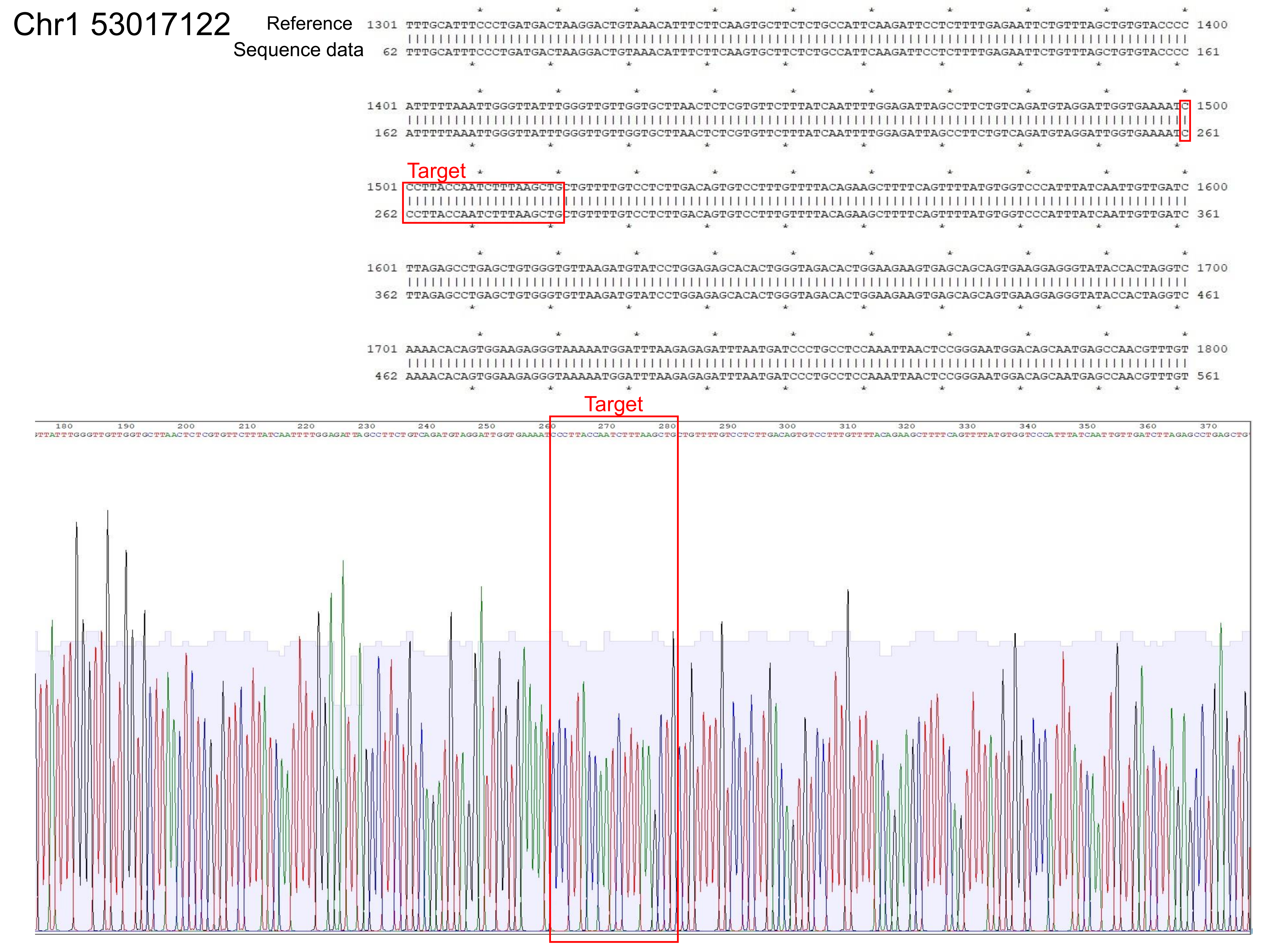
Chr1 53017122
Reference
Sequence data
Target
Target

Chr1 61419661

```
Reference       1401 CTGAGAGGAAAGAAGGGCTCCGGAAGGGGAGGGACTCCGGAAACACTGACCCAAAAGTGAAAAGCAAATCCCGCCATCCAGCCAGCCTAGGGCGGCAGAA 1500
                     ||||||||||||||||||||||||||||||||||||||||||||||||||||||||||||||||||||||||||||||||||||||||||||||||||||
Sequence data    126 CTGAGAGGAAAGAAGGGCTCCGGAAGGGGAGGGACTCCGGAAACACTGACCCAAAAGTGAAAAGCAAATCCCGCCATCCAGCCAGCCTAGGGCGGCAGAA 225

                1501 AATACGGGACAGATACATAGTTAATGAGGGAAAGGAAGGGGCGGAAATGAGAAAGAAAAGGGTCTGCATATGAATAAAATGATACACGGTGTCTGTAATC 1600
                     ||||||||||||||||||||||||||||||||||||||||||||||||||||||||||||||||||||||||||||||||||||||||||||||||||||
                 226 AATACGGGACAGATACATAGTTAATGAGGGAAAGGAAGGGGCGGAAATGAGAAAGAAAAGGGTCTGCATATGAATAAAATGATACACGGTGTCTGTAATC 325

                                                                                                     Target
                1601 CCTCAGGATTGGTTCTCTGCACCTCTTAACCAAAGGCAACAATGAAAAGCAAGCCTCGCTACACAGGGAGAAGGGCCCTTTACCACCTGAAGAGCTCAGA 1700
                     ||||||||||||||||||||||||||||||||||||||||||||||||||||||||||||||||||||||||||||||||||||||||||||||||||||
                 326 CCTCAGGATTGGTTCTCTGCACCTCTTAACCAAAGGCAACAATGAAAAGCAAGCCTCGCTACACAGGGAGAAGGGCCCTTTACCACCTGAAGAGCTCAGA 425

                1701 TAAACAAATCAAGTTGAAGAAATTTACCTTTCTCCCTTCTCCTACAAAGATCCTGGGTTTTTGTTTGTTTGTTTTTGGTTTTTCGAGACATGGTTTCTCT 1800
                     ||||||||||||||||||||||||||||||||||||||||||||||||||||||||||||||||||||||||||||||||||||||||||||||||||||
                 426 TAAACAAATCAAGTTGAAGAAATTTACCTTTCTCCCTTCTCCTACAAAGATCCTGGGTTTTTGTTTGTTTGTTTTTGGTTTTTCGAGACATGGTTTCTCT 525

                1801 GTATAGCCCTGGCTGTCCTGGAACTCACTCTGTAGACCAGGCTGGCCAGGAACTCAGAAATCCGCCTGCCTCTGCCTCCCAAGTGCTGGGATTAAAGGCG 1900
                     ||||||||||||||||||||||||||||||||||||||||||||||||||||||||||||||||||||||||||||||||||||||||||||||||||||
                 526 GTATAGCCCTGGCTGTCCTGGAACTCACTCTGTAGACCAGGCTGGCCAGGAACTCAGAAATCCGCCTGCCTCTGCCTCCCAAGTGCTGGGATTAAAGGCG 625
```

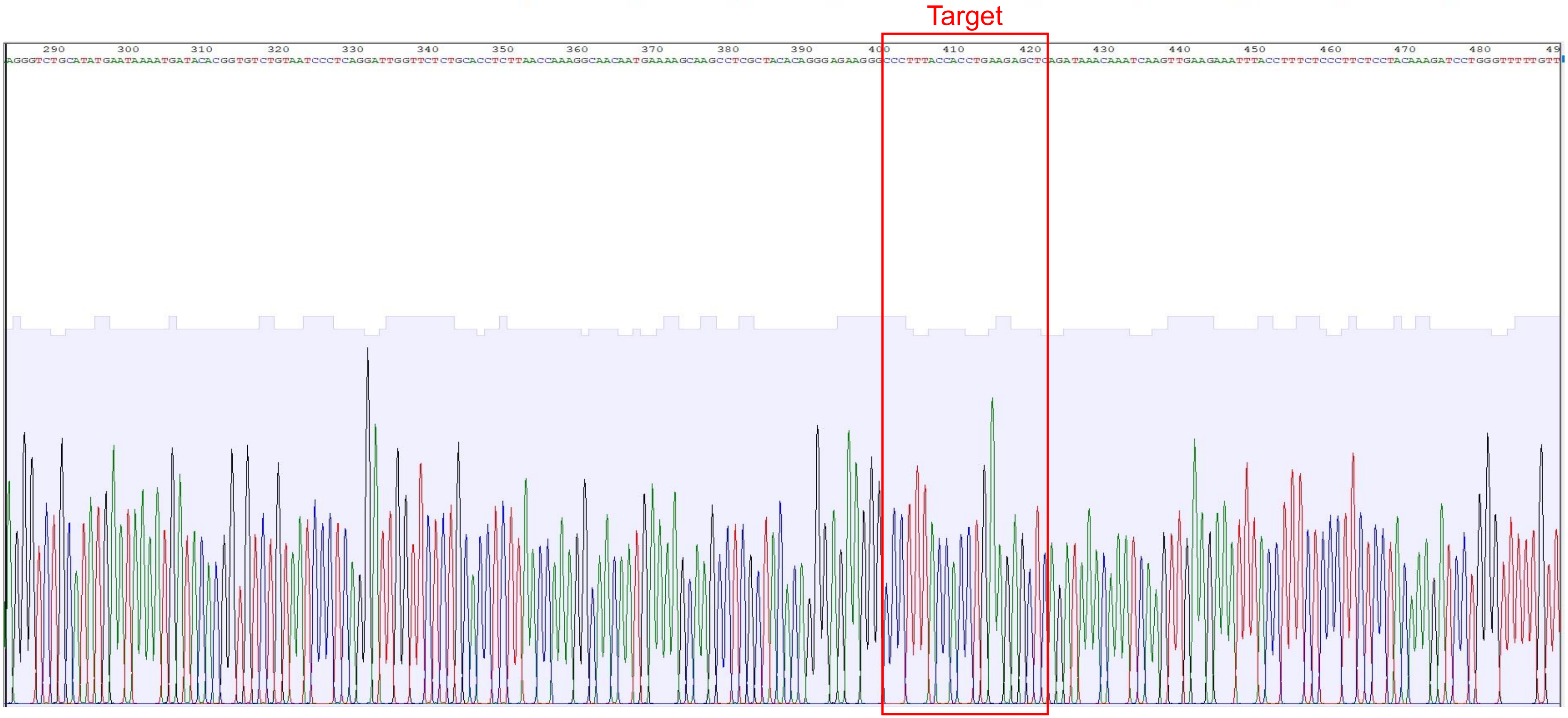

Chr3 104555268

Reference / Sequence data

```
1601 CCCTTGCCTTGGTTTATTTCTTCAGTCACGCACCTCCTTTCTCCCCTCTGGTTACGTGCTGTGTGCGTACCTGCCCAGTGACTTCTCCCTGAAGCATCGC 1700
     ||||||||||||||||||||||||||||||||||||||||||||||||||||||||||||||||||||||||||||||||||||||||||||||||||||
 275 CCCTTGCCTTGGTTTATTTCTTCAGTCACGCACCTCCTTTCTCCCCTCTGGTTACGTGCTGTGTGCGTACCTGCCCAGTGACTTCTCCCTGAAGCATCGC 374

1701 CTGATGCTGATGAATGGCTGTGTAAGCAGGCAGGTGTGAGGCTTAATTACCACTGGCTGCCACAGGCCTATTTTCATTCTTCAGTGGGGCTGGGCAGTGT 1800
     ||||||||||||||||||||||||||||||||||||||||||||||||||||||||||||||||||||||||||||||||||||||||||||||||||||
 375 CTGATGCTGATGAATGGCTGTGTAAGCAGGCAGGTGTGAGGCTTAATTACCACTGGCTGCCACAGGCCTATTTTCATTCTTCAGTGGGGCTGGGCAGTGT 474

          Target
1801 TAAGACCCTTAACATTCTTGGAGCTCATGTGTGTCTGGACTGTGGAGTGGGCTGGAGGAGATGACAAGAGGAAAGTTTAGATAAATGGTTCATCTGGGGA 1900
     ||||||||||||||||||||||||||||||||||||||||||||||||||||||||||||||||||||||||||||||||||||||||||||||||||||
 475 TAAGACCCTTAACATTCTTGGAGCTCATGTGTGTCTGGACTGTGGAGTGGGCTGGAGGAGATGACAAGAGGAAAGTTTAGATAAATGGTTCATCTGGGGA 574

1901 TGAACCGGCACAGCAGCTAAGGAGGCCGAGGCAGAAGAATTGTTTTGAGTTCCAATCGAGCTCCGTAATGAAAGTCAGGCCAGCAATGAGATCCTATTTC 2000
     ||||||||||||||||||||||||||||||||||||||||||||||||||||||||||||||||||||||||||||||||||||||||||||||||||||
 575 TGAACCGGCACAGCAGCTAAGGAGGCCGAGGCAGAAGAATTGTTTTGAGTTCCAATCGAGCTCCGTAATGAAAGTCAGGCCAGCAATGAGATCCTATTTC 674

2001 AAAAGTTCTAAAGCCGGGCAGTGGTGGCACACGCCTTTAATCCCAGCACTTGGGAGGCAGAGGCAGGCGGATTTCTGAGTTCGACACCAGCCTGGTCTAC 2100
     ||||||||||||||||||||||||||||||||||||||||||||||||||||||||||||||||||||||||||||||||||||||||||||||||||||
 675 AAAAGTTCTAAAGCCGGGCAGTGGTGGCACACGCCTTTAATCCCAGCACTTGGGAGGCAGAGGCAGGCGGATTTCTGAGTTCGACACCAGCCTGGTCTAC 774
```

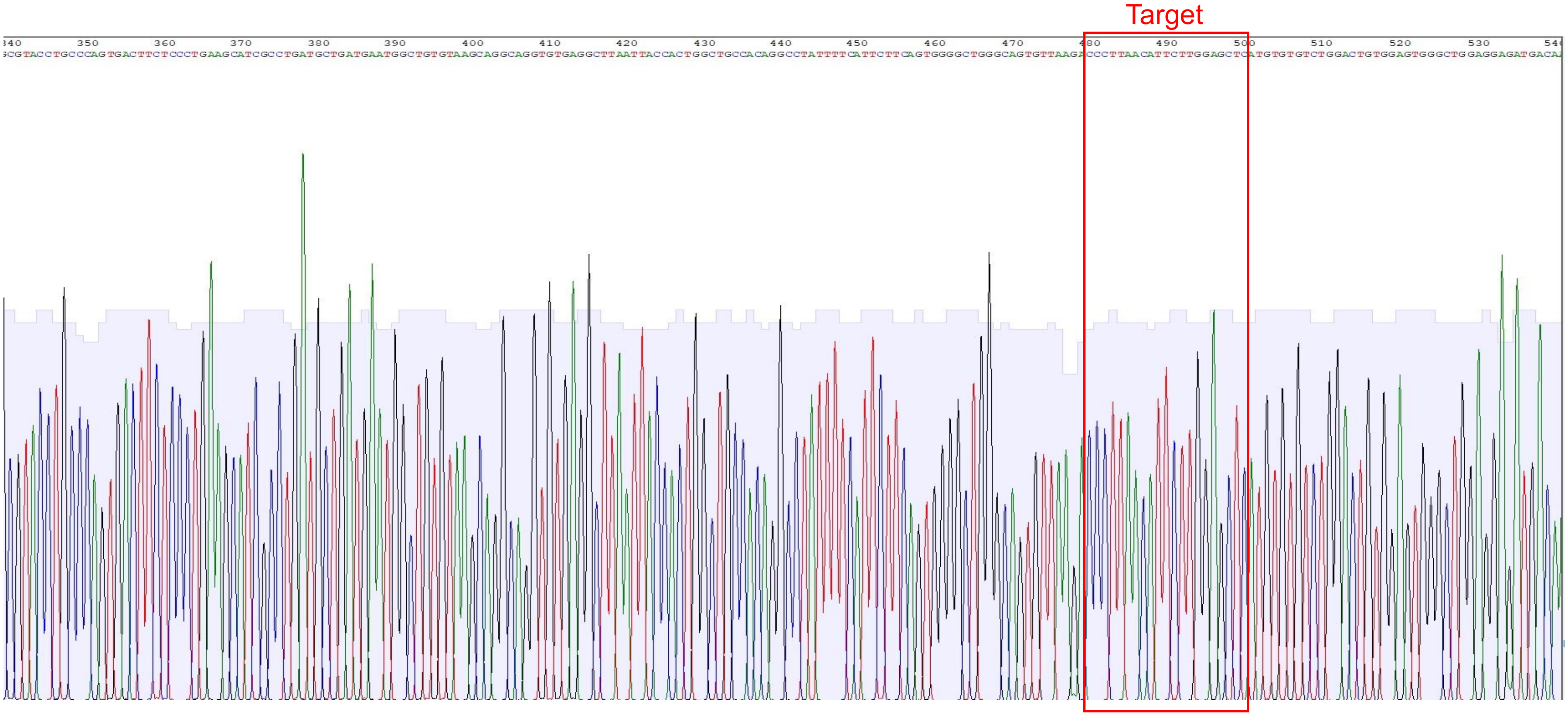

Chr3 108811978

```
                  1601 AAGGAGGGAGATGGACAAATAATGTCAGGGCTGAGACGTCACATCTCTATAGCTACAGAGTAAAGGACAGAACTATCCTAGTCTAGAAGAGGAGGTGGCC 1700
Reference              ||||||||||||||||||||||||||||||||||||||||||||||||||||||||||||||||||||||||||||||||||||||||||||||||||||
Sequence data     53   AAGGAGGGAGATGGACAAATAATGTCAGGGCTGAGACGTCACATCTCTATAGCTACAGAGTAAAGGACAGAACTATCCTAGTCTAGAAGAGGAGGTGGCC 152

                  1701 AGGGAACCATCGACTGGCAGTGCCGTGCAGTAGGTGGCTTGAAAGGAGTATACCGAATATAGTGGCAGCACAGGCTAGGATTACCTTACCTGAACTAGAC 1800
                       ||||||||||||||||||||||||||||||||||||||||||||||||||||||||||||||||||||||||||||||||||||||||||||||||||||
                  153  AGGGAACCATCGACTGGCAGTGCCGTGCAGTAGGTGGCTTGAAAGGAGTATACCGAATATAGTGGCAGCACAGGCTAGGATTACCTTACCTGAACTAGAC 252

                                                   Target
                  1801 CTGGAAAGCCTCACAGGACGAGGCCCGTGAGCTTCAAGAATGGTGAGGGCATAATAAAGGGAGTTTTTGCAAGAGTAGCAGGAGTCTGAGTAGAGACATA 1900
                       ||||||||||||||||||||||||||||||||||||||||||||||||||||||||||||||||||||||||||||||||||||||||||||||||||||
                  253  CTGGAAAGCCTCACAGGACGAGGCCCGTGAGCTTCAAGAATGGTGAGGGCATAATAAAGGGAGTTTTTGCAAGAGTAGCAGGAGTCTGAGTAGAGACATA 352

                  1901 GAAAGGTAAAATTCAGTATTAAACAACGTGGATGGAAGATGTCAATGTAGATGCAGTGCTGGTTACTTGAAAAGTTAGTGACTTTTGGCTTAGAGGCCTA 2000
                       ||||||||||||||||||||||||||||||||||||||||||||||||||||||||||||||||||||||||||||||||||||||||||||||||||||
                  353  GAAAGGTAAAATTCAGTATTAAACAACGTGGATGGAAGATGTCAATGTAGATGCAGTGCTGGTTACTTGAAAAGTTAGTGACTTTTGGCTTAGAGGCCTA 452

                  2001 ATGAAAGCTATTATTCTGTTAGAAAATAGAATGGCTTAATAAACATAGTGGGCAGTGAATGGAAATATTTGTTAAGGCAAAGAATGATAATATCTCTTTA 2100
                       ||||||||||||||||||||||||||||||||||||||||||||||||||||||||||||||||||||||||||||||||||||||||||||||||||||
                  453  ATGAAAGCTATTATTCTGTTAGAAAATAGAATGGCTTAATAAACATAGTGGGCAGTGAATGGAAATATTTGTTAAGGCAAAGAATGATAATATCTCTTTA 552
```

Target

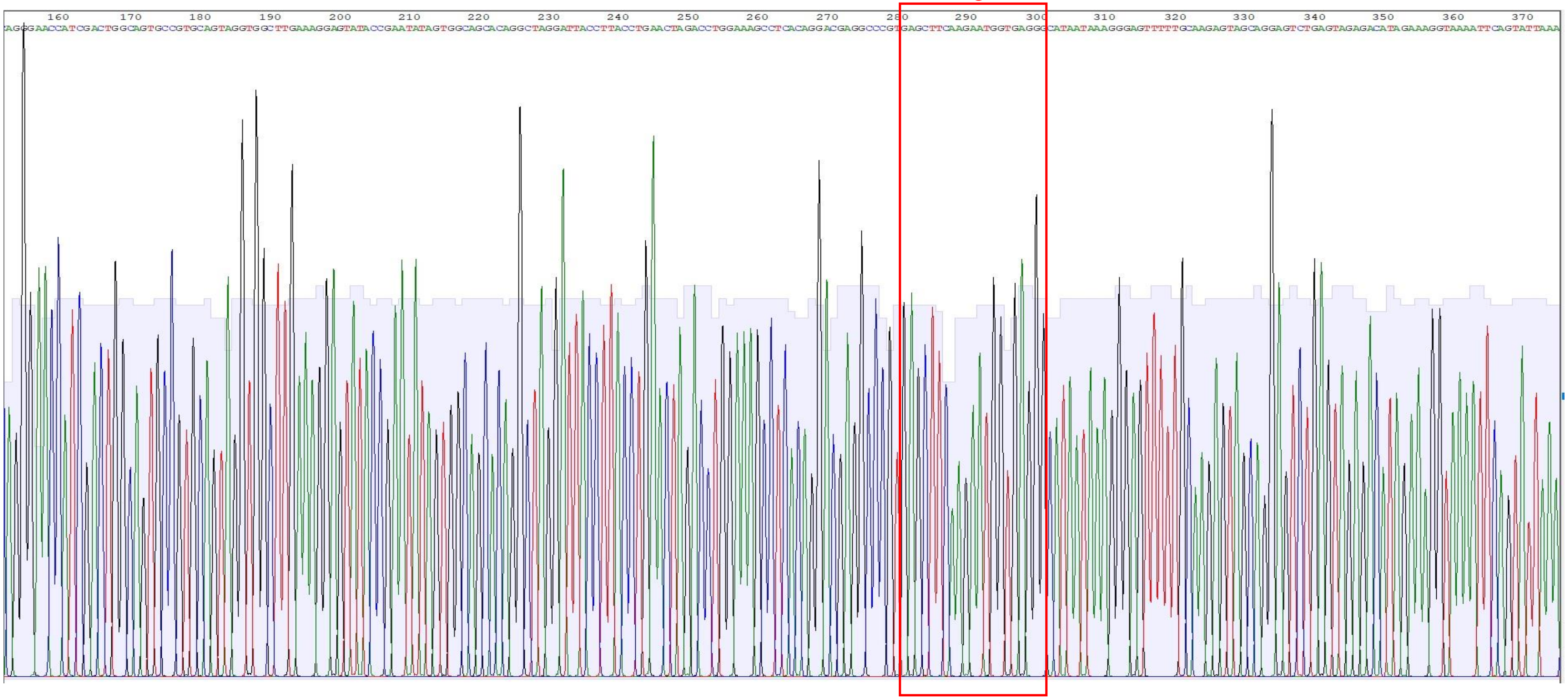

Chr4 87053729

```
Reference       2301 GTGGTACTTGCAATGATGACGGCTTCTCGAATTCTTCCCTGTGTGGGTCATCCTGTATGCTCTTCACTGCTACTCTGTTTATAGGACAGAGCAATAACCA 2400
                     ||||||||||||||||||||||||||||||||||||||||||||||||||||||||||||||||||||||||||||||||||||||||||||||||||||
Sequence data    109 GTGGTACTTGCAATGATGACGGCTTCTCGAATTCTTCCCTGTGTGGGTCATCCTGTATGCTCTTCACTGCTACTCTGTTTATAGGACAGAGCAATAACCA 208

                2401 TGCTGAGGATGCCAGCATTCAGAGAGAGGAAACACCTTGCTCAGAGTCACATGTCCCATAAGCAGTACATCTTGGTTCCCAAACTACACCTCCAATTTCA 2500
                     ||||||||||||||||||||||||||||||||||||||||||||||||||||||||||||||||||||||||||||||||||||||||||||||||||||
                 209 TGCTGAGGATGCCAGCATTCAGAGAGAGGAAACACCTTGCTCAGAGTCACATGTCCCATAAGCAGTACATCTTGGTTCCCAAACTACACCTCCAATTTCA 308

                                                                                          Target
                2501 AATCCCATCTTCTTCCATGCACTTTATGCTGAACATAATCCTTCCATGCAAGCCAGTACTCACCCATACATCTTGACGAGCACCAGACAACAGCAGTTGC 2600
                     ||||||||||||||||||||||||||||||||||||||||||||||||||||||||||||||||||||||||||||||||||||||||||||||||||||
                 309 AATCCCATCTTCTTCCATGCACTTTATGCTGAACATAATCCTTCCATGCAAGCCAGTACTCACCCATACATCTTGACGAGCACCAGACAACAGCAGTTGC 408

                2601 TTAACTGCAGTTTGGACTGCAATCAGGACTAAGAAGGCCTTGATTTTTCAGATTGTCCAGAAAAGGTCAAACCCATACCCAGAAATAAAAGCTAGATCTT 2700
                     ||||||||||||||||||||||||||||||||||||||||||||||||||||||||||||||||||||||||||||||||||||||||||||||||||||
                 409 TTAACTGCAGTTTGGACTGCAATCAGGACTAAGAAGGCCTTGATTTTTCAGATTGTCCAGAAAAGGTCAAACCCATACCCAGAAATAAAAGCTAGATCTT 508

                2701 TGAAAAGAAGAGAACGTCAATCATTAATTTTTCCCATGAGATGGCACTTAAATAATAAATGTGTATTCGCTTTTGTTTCTTCACCTCCAGTTCTCATTCA 2800
                     ||||||||||||||||||||||||||||||||||||||||||||||||||||||||||||||||||||||||||||||||||||||||||||||||||||
                 509 TGAAAAGAAGAGAACGTCAATCATTAATTTTTCCCATGAGATGGCACTTAAATAATAAATGTGTATTCGCTTTTGTTTCTTCACCTCCAGTTCTCATTCA 608
```

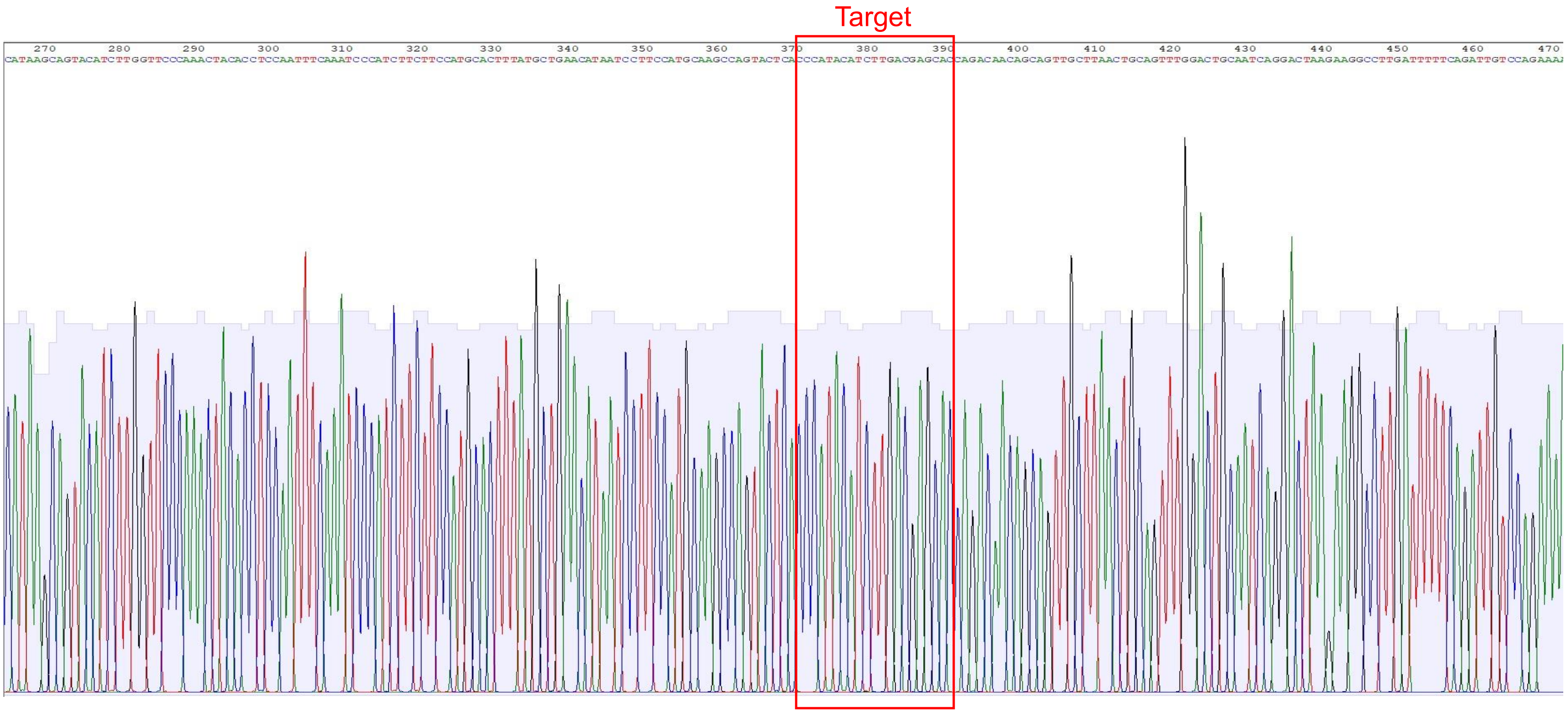

Chr7 135695712

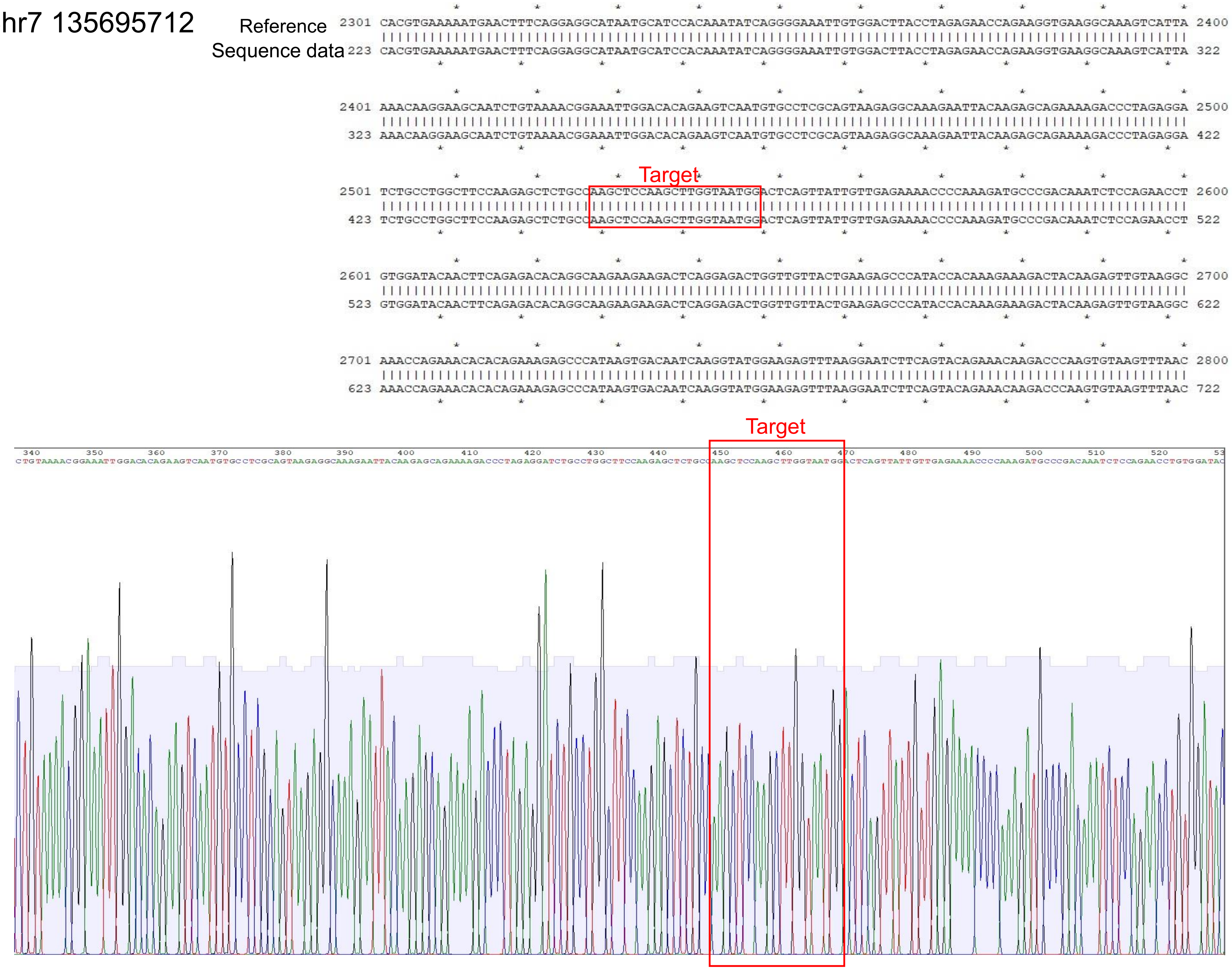

Supplement: Supplementary file 3 — Supplementary Material 3 [file 13041_2025_1240_MOESM3_ESM.pdf]
